# Sustainable analysis of COVID-19 Co-packaged paxlovid: exploring advanced sampling techniques and multivariate processing tools

**DOI:** 10.1186/s13065-025-01567-2

**Published:** 2025-07-10

**Authors:** Shymaa S. Soliman, Nisreen F Abo- Talib, Mohamed R. Elghobashy, Mona A. Abdel Rahman

**Affiliations:** 1 Analytical Chemistry Department, Faculty of Pharmacy, October 6 University, October 6 City, Giza 12858 Egypt; 2 Egyptian Drug Authority, P.O. Box 35521, Agouza, Giza Egypt; 3 Analytical Chemistry Department, Faculty of Pharmacy, Cairo University, El-Kasr- El Aini Street, Cairo, 11562 Egypt

**Keywords:** COVID-19 Co-packaged Paxlovid^®^, Latin hypercube sampling, Multivariate chemometric models, Sustainability assessment tools, New hybrid GA-ICOMP-PLS method

## Abstract

The drawbacks of random sampling not only hinder the development of more reliable and efficient methods but also weaken their accuracy, predictive abilities, and validity across several domains. During the current study, a pioneering statistical technique namely, Latin Hypercube Sampling (LHS) was integrated with different multivariate chemometric models namely; Partial Least Squares (PLS), Genetic Algorithm‑Partial Least Squares (GA-PLS), Artificial Neural Networks (ANN), and Multivariate Curve Resolution‑Alternating Least Squares (MCR-ALS). This integration aimed to achieve full data coverage and thereby enhance the predictive powers of these models. Being of clinical significance, Paxlovid^®^, a newly co-packaged antiCOVID-19 drug containing ritonavir (RNV)-boosted nirmatrelvir (NMV), was utilized as a study subject to demonstrate the powerful potentials of LHS in enhancing models’ robustness and predictive accuracy. The LHS technique was able to provide well-interpreted and informative samples by capturing essential variabilities across the input space without any increase in sample numbers. It was compared and outperformed the random sampling Monte Carlo technique. A comprehensive comparison between the developed models was held where the RMSEP was relatively reduced by 14.1%, 8.9%, 53.1%, and 34.6% for RNV and NMV, respectively using the ANN and MCR-ALS models. Various preprocessing techniques were employed to improve signal quality for PLS construction, yielding superior results (RMSEC of 0.19 for both RNV and NMV) compared to the original, unprocessed spectral data (RMSEC of 0.21 for both RNV and NMV). The Principal Component Analysis score plot was constructed, confirming the consistency of the dataset and the absence of systematic errors, enhancing confidence in the models’ robustness. A new hybrid variable selection strategy (GA-ICOMP-PLS) was developed to enhance the robustness and parsimony of the GA-PLS model. Prediction error values of 0.15 and 0.14 were successfully achieved for RNV and NMV, respectively, indicating strong predictive power and generalization. Consistent with sustainability and eco-friendly goals, the current study pioneers the usage of green–blue-white alternatives to conventional analytical methods. A comprehensive assessment was conducted using the “Sample Preparation Metric of Sustainability”, the “Analytical Greenness metric for Sample Preparation” and the “Analytical Greenness metric” alongside two solvent sustainability evaluation tools. These evaluations yielded promising results, with green quadrant classification and high scores of 5.89, 0.67, and 0.82 for each metric, respectively, as well as satisfactory t- and F-test values. Moreover, the models achieved outstanding results on the RGB12 metric and Blueness Applicability Grade Index, scoring 96.8% and 82.5, respectively, highlighting their broad applicability, high efficiency, and alignment with eco-friendly analytical practices.

**Supplementary Information:**

The online version contains supplementary material available at 10.1186/s13065-025-01567-2.

## Introduction

Random sampling techniques often suffer from significant constraints regarding data quality and reliability leading to serious gaps in sampling and data duplications [1]. Methods relying on such techniques frequently experience several drawbacks such as robustness absence, biased performance, and restrained predictive abilities [2]. Therefore, compensating for these limitations usually necessitates the usage of various datasets, causing serious environmental impacts including high waste generation, extensive resource utilization, and increased expenses.

Latin Hypercube Sampling (LHS) stands out as a robust schematic statistical technique that effectively navigates multidimensional sample spaces, producing representative data points [3]. Unlike random sampling, LHS ensures uniform coverage of each sample by understanding complex systems and capturing essential variabilities without any increase in sample numbers. It acts as a solid indispensable tool for developing innovative consistent methods with enhanced performance, optimized experimental designs, increased ecological activity, and improved data interpretation [4]. LHS mainly depends on dividing sample space into equal intervals or strata then each sample is distributed randomly at least one time to avoid biases and sample clustering within certain areas, ensuring good representation and full contribution of all variables [5]. The latter is independently stratified to evade the influence of multiple-dimensional sampling, thereby reducing data redundancy and enhancing variable space coverage making LHS useful in different analytical fields including environmental modeling [6], engineering design [7], financial risk analysis [8], medical research [9] and manufacturing processes [10].

Chemometrics is one of these techniques that play a critical role in interpreting complex data, optimizing analytical processes, and improving predictions’ accuracy across a wide range of disciplines. It depends on extracting profound information from multivariate data which helps to discern inapparent patterns and relationships between different variables [11]. Commonly used models such as Partial Least Squares (PLS), Genetic Algorithm‑Partial Least Squares (GA-PLS), Artificial Neural Networks (ANN), and Multivariate Curve Resolution-Alternating Least Squares (MCR-ALS), are extensively utilized in various disciplines, including pharmaceuticals, ecological studies, agriculture, and food quality analysis [12–15]. However, despite their widespread utility, these models often underperform in real-world applications, unfulfilling the sustainability goals of method consistency and resource efficacy. This may be attributed to their reliance on random data partitioning during model calibration which can lead to inadequate sample representation and inflated accuracy estimates. Thus, following more systematic sampling strategies such as the LHS technique is essential to provide samples with better interpretation and comprehensive coverage. Moreover, the accuracy and robustness of these models can be usually influenced by high-frequency noise accompanied by the UV spectral data. Thus, employing signal preprocessing techniques such as the Savitzky-Golay filter, spectral derivatization, and Standard Normal Variate (SNV) smoothing is sometimes necessary to remove irrelevant variations while maintaining the relevant analytical signals. These preprocessing procedures are particularly valuable in multivariate calibration approaches such as the PLS and the Principal Component Analysis (PCA) models, where the quality of input data directly influences the model’s predictive power. This combination improves not only the signal-to-noise ratio but also ensures the optimal relevancy and accurate interpretation of the extracted features towards the subsequent modeling.

Despite the importance of centralized techniques in various analytical disciplines, they may not always be suitable for every organization or situation. Investigating greener alternatives and automation solutions may help alleviate some of their challenges [12]. In modern analytical techniques, the concepts of Green Analytical Chemistry (GAC) and White Analytical Chemistry (WAC) are contemporarily applied to achieve a comprehensive equivalence between method greenness, performance, practicality, and cost-efficiency [16, 17]. The GAC identifies analytical processes and areas that need improvement such as energy consumption, reagents, and solvents usage to reduce the environmental hazards of implemented methods [16]. Moreover, the WAC emphasizes resource efficiency by pinpointing opportunities to optimize resource usage and minimize waste [17]. A recent concept named “Blueness” [18] has been introduced as a complementary metric to the existing green and whiteness principles. It focuses mainly on the methods’ practicality where the blue color is inspired by the Red-Green-Blue model (RGB). Despite the environmental evaluation and analytical sustainability offered by these metrics, they possess certain limitations. They often rely on semi-quantitative assessments or predefined scoring systems that may not fully capture the complexity of analytical performance, especially in terms of selectivity, matrix effects, or method ruggedness [19, 20]. The relative weighting of parameters in such tools can introduce subjectivity. Similarly depending on predefined principles may not adequately account for method selectivity. Therefore, while these metrics are valuable screening tools offer a valuable framework for comparative assessment and visual representation, their scores should be interpreted in conjunction with conventional validation parameters and expert judgment.

**Fig. 1:**
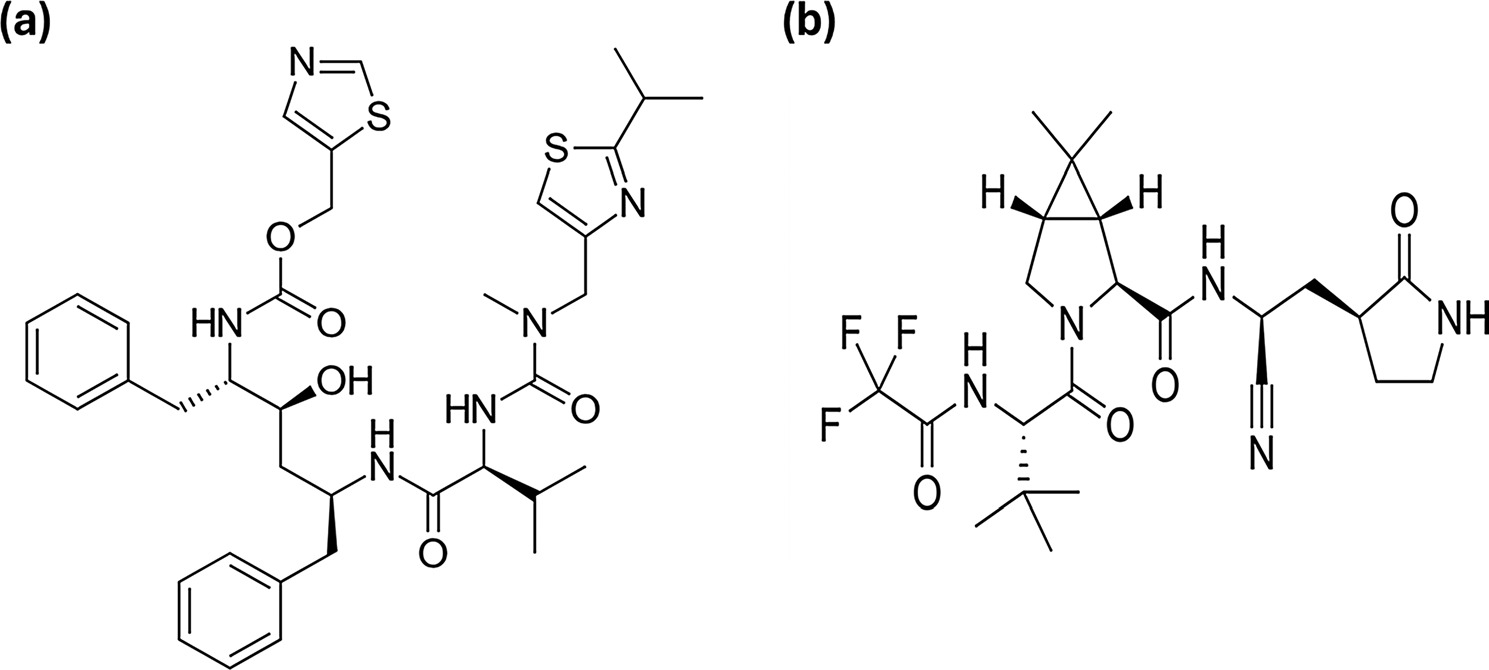
Structure of Paxlovid^®^ co-packaged drugs; (**a**) ritonavir and (**b**) nirmatrelvir

Ritonavir (RNV), known as 1,3-thiazol-5-ylmethyl N-[(2 S,3 S,5 S)-3-hydroxy-5-[[(2 S)-3-methyl-2-[[methyl-[(2-propan-2-yl-1,3-thiazol-4-yl)methyl] carbamoyl]amino]butanoyl]amino]-1,6-di(phenyl)hexan-2-yl]carbamate (Fig. 1a), is a protease and potent CYP3A inhibitor used for treating human immunodeficiency virus type 1 (HIV-1) by interfering with the virus reproductive cycle. Recently, RNV revealed valuable boosting effects in combined regimens such as Paxlovid^®^. The latter is a newly co-packaged antiCOVID-19 drug containing RNV and nirmatrelvir (NMV) as the main active constituents. NMV, known as (1R,2 S,5 S)-N-[(1 S)-1-cyano-2-[(3 S)-2-oxopyrrolidin-3-yl]ethyl]-3-[(2 S)-3,3-dimethyl-2-(2,2,2-trifluoroacetamido)butanoyl]-6,6-dimethyl-3-azabicyclo[3.1.0]hexane-2-carboxamide (Fig. 1b), is another protease inhibitor that is specifically target the COVID-19 virus through the attack of its protease enzyme and polyproteins, preventing viral replication within human cells [21]. Although RNV does not directly exhibit antiviral activity against the COVID-19 virus, it enhances the therapeutic effectiveness of NMV by inhibiting the CYP3A4 enzyme responsible for NMV metabolism, thereby increasing NMV’s plasma concentration and half-life. Within the past five years, different analytical methods were reported for assaying RNV or NMV either alone or in different mixtures. RNV was determined alone [22–24] and in combination with other drugs or impurities [25–31] using different chromatographic and spectrophotometric methods. While NMV was determined in different matrices including bulk [32, 33], dosage form [34], blood [35], and degradation products [36–38]. Additionally, various techniques were published regarding RNV and NMV determination in plasma and their co-packed tablets such as high-performance liquid chromatography (HPLC) [39–41], liquid chromatography-tandem mass spectrometry (LC-MS/MS) [42–46], ultra-performance liquid chromatography-tandem mass spectrometry (UPLC-MS/MS) [47–49], high-performance thin-layer chromatography (HPTLC) [50], micellar electrokinetic chromatography [51], spectrofluorometry [52] and spectrophotometry [53].

During the current work cost-effective, sustainable, and robust multivariate chemometric models; PLS, GA-PLS, ANN, and MCR‑ALS were developed for selectively assaying the co-packaged drugs; RNV and NMV, in their bulk, Paxlovid^®^ formulation and human plasma. Using various models for the analysis is recommended to guarantee data suitability, robustness, comprehensiveness, and consistency. The implemented models are known for their complementary strengths in handling spectral overlap and nonlinearities. The key rationale behind our approach lies in recognizing that sampling strategy is a frequently overlooked yet critical aspect, affecting the robustness and predictive accuracy of chemometric models. In this context, we aimed to assess the influence of the LHS technique on the models’ predictive power as we hypothesized that implementing LHS, a space-filling and stratified design, would significantly enhance the generalizability, accuracy, reliability, predictive performance, and robustness of the developed models by ensuring uniform data coverage across the experimental domain. A comparative analysis against the widely used Monte Carlo (MC) sampling technique was conducted to further validate our hypothesis and assess the effectiveness of LHS. Additionally, a new hybrid technique namely; genetic algorithm information complexity PLS (GA-ICOMP-PLS) was developed to enhance the robustness of the constructed models. Various signal preprocessing techniques such as the Savitzky-Golay filter, spectral derivatization, and SNV smoothing were integrated with LHS, providing an advanced modeling framework for spectroscopic data interpretation. PCA score plot was obtained to further assess the homogeneity of the spectral data for multivariate calibration. A greenness evaluation using the “Sample Preparation Metric of Sustainability” (SPMS), the “Analytical Greenness metric for Sample Preparation” (AGREEprep), and the “Analytical Greenness metric” (AGREE) in addition to “whiteness” and “blueness” estimations using the RGB12 and the Blue Applicability Grade Index (BAGI) tools were then followed to evaluate the models’ practicality and sustainability. Despite the enriched literature on Paxlovid^®^ determination, no methods have incorporated the LHS technique or signal preprocessing techniques with multivariate UV spectrophotometry during their study. As far as we know, the developed study was the first to introduce the straightforward LHS methodology and signal preprocessing tools for the simultaneous determination of RNV and NMV in different matrices. The outcomes of this work will provide valuable insight into the role of systematic sampling in multivariate calibration. Specifically, it will: (i) highlight the influence of LHS on prediction accuracy and error reduction compared to random techniques, (ii) offer comparative performance metrics such as RMSEC and RMSEP across multiple models, (iii) propose practical guidelines for integrating LHS in chemometric workflows for spectroscopic applications, (iv) establish the novelty of combining LHS with chemometric modeling for simultaneous drug analysis in pharmaceutical and biological matrices, (v) validate the integration of various signal preprocessing techniques in improving prediction accuracy and spectral resolution, and (vi) support the method’s environmental and analytical sustainability using greenness (AGREE, AGREEprep, SPMS), whiteness (RGB12), and blueness (BAGI) metrics.

## Materials and methods

### Instrumentation

A dual beam UV-visible spectrophotometer (Shimadzu 1800) was implemented for spectrophotometric measurements using a quartz cell of 1.0 cm path length. The software of Shimadzu UV Probe 2.32 was used for data acquisition. A single scanning mode with a slit width of 1.0 nm and sampling interval of 0.2 nm was utilized during the study. MATLAB^®^ software (version 8.1.0.604) was used for data manipulation and model construction using the PLS and MCR-ALS toolboxes. LHS sampling was carried out using built-in scripts found in MATLAB^®^ R2013a for samples’ stratification.

### Reagents and samples

- Ethanol, acetonitrile, and methanol of HPLC-grades were obtained from Sigma-Aldrich (Germany). Bi-distilled water was incorporated during the work.
- Pure samples of RNV (99.0%), NMV (99.0%), and Paxlovid^®^ medication (Batch No: 220030) were provided by Pfizer, Inc., Egypt. The medication contains oval pink-coated tablets of NMV (150.0 mg) co-packaged with white-coated tablets of RNV (100.0 mg).

### Standard solutions

All solutions were formulated based on the spectral intensity and the proportion of each component in the final mixtures. Appropriate dilutions of the mixed portions were ensured to a total volume of 10 mL.

#### Standard stock solutions

Different solutions of RNV and NMV (1.0 mg mL^− 1^) were accurately prepared by weighing 100.0 mg of each component and then separately transferred into 100 mL volumetric flasks and dissolved in 80 mL ethanol followed by 10 min sonication and volume completion to the mark using the same solvent (ethanol).

#### Working standard solutions

The desired working ranges were prepared using ethanol through the appropriate dilution of the stock solutions. A concentration value of 100.0 µg mL^− 1^ was separately obtained for both drugs from their subsequent stock solutions. Certain aliquots were accurately transferred into 100 mL volumetric flasks and the volumes were adjusted to the mark using ethanol.

### Procedures

#### Construction and validation of calibration models

A multilevel multifactorial design of five different concentration levels (-2, -1, 0, 1, and 2) was followed for each component to successfully construct various mixtures with even representations. Twenty-five mixtures containing concentration ranges of 5.0 to 25.0 µg mL^− 1^ for each drug were successfully prepared and used as calibration or training sets. The mixtures’ absorption spectra were obtained within a range of 190–400 nm using 0.2 nm intervals and ethanol as blank. A measuring range of 200–260 nm was employed for accurate data acquisition. The spectra were later exported to the MATLAB^®^ program (R2013a), enhanced, mean-centered, and processed using the adopted models including PLS, GA-PLS, ANN, and MCR-ALS. The models were fine-tuned using the ideal number of latent variables (LVs) for PLS, variable configurations for GA-PLS, Plackett–Burman design for ANN, and non-negativity constraints for MCR-ALS.

Different validations were carried out to estimate the predictive power of the constructed models. Various mixtures containing well-distributed random concentrations and used as a validation set. The latter was prepared using the LHS strategy to guarantee descriptive sampling of the concentration region, thus enabling consistent validation of the model. The concentrations were stratified into 13 equal probability intervals using built-in scripts found in MATLAB^®^ R2013a to acquire better variability and a more accurate model. The suggested mixtures were prepared using the same procedures as the calibration set, ensuring comprehensiveness and a balanced dataset for both the training and validation phases. The spectra were integrated using the established wavelength range, mean-centered, and processed using the structured models to value their predictive powers.

#### Optimization of the chemometric models

Savitzky-Golay filter, spectral derivatization, and SNV transformation techniques were employed as signal preprocessing tools to enhance and improve the analyzed spectral data. Savitzky-Golay smoothing was applied using a third-order polynomial and an eleven-point frame-length window to smooth the raw spectra. These parameters were selected based on literature preference and visual inspection of noise reduction without significant signal distortion. Subsequently, both first and second-order derivatives were computed to enhance spectral resolution. The preprocessed data were then normalized using the SNV transformation to minimize scatter and path length effects. This combination of preprocessing steps improved baseline stability and allowed better separation of analyte signals in the multivariate analysis thus preventing arbitrary signal manipulation. Afterward, PCA was conducted to evaluate the internal structure of these spectra and assess their fitness and suitability for multivariate calibration. The absorbance data was collected as a single matrix and subjected to the existing PCA function. A score plot was generated using the first two principal components (PC1 and PC2), illustrating sample distribution and potential outliers. Samples were color-coded based on total drug concentrations and differentiated by marker edge color into two sets. This step ensured data compliance, quality, and structural consistency with the requirements of chemometric models. The models were then built using absorbance data collected in the 200–260 nm range, where both drugs displayed distinguishable and quantifiable spectral features.

The PLS model, a widely used linear regression technique, was then constructed using both calibration and validation datasets. Mean-centering method was applied to improve data interpretation while model optimization was carried out via leave-one-out cross-validation, where the root mean square error of cross-validation (RMSECV) was used to determine the optimal number of LVs, achieving a balance between accuracy and model simplicity. The GA-PLS tool was used to boost the predictive performance of the standard PLS model by selecting the most informative wavelength variables. The GA tool optimized variable selection through simulated evolutionary steps such as crossover, mutation, and selection. Model tuning was performed using 25 calibration spectra, with the GA parameters (e.g., population size, mutation rate, and subset size) adjusted to identify the best-performing subset of wavelengths from the full range of 301 variables. Over multiple generations, the algorithm refined the variable subset by prioritizing wavelengths contributing to improved predictive accuracy. Two enhancements such as penalty terms and multiple GA runs were conducted to address the susceptibility of the GA to local optimization. Additionally, the ANN model was implemented for its strong capability in nonlinear modeling and recognizing complex patterns within data. The model was trained using backpropagation and refined through iterative weight adjustments to minimize prediction error. A sigmoid and Purelin-to-Purelin transfer function was used in both the hidden and output layers to support model flexibility and performance. The MCR-ALS model was applied to resolve overlapping spectral signals and extract pure component information from complex mixtures. Non-negativity constraints were imposed on both the spectral and concentration profiles, ensuring realistic and interpretable results. These constraints also helped minimize iteration count and improve convergence efficiency.

#### Evaluation of training and testing datasets

The consistency and true accuracy of the constructed models were fully assessed by calculating reliable parameters such as the recovery percent (% R), standard deviation (SD), relative standard deviation (% RSD), relative error (% RE), and correlation coefficient value (r). The models’ performance was evaluated using both the root mean square error of calibration (RMSEC) and the RMSECV. The RMSEC focuses on assessing the fitness of the models with the training data. While the RMSECV offers different insights into models’ generalization towards unseen data. Moreover, the root mean square error of prediction (RMSEP) was used for assessing the models’ exactness and reliability toward validation set prediction. It evaluates the practical performance of the constructed models toward new or unseen data by using an independent validation set. Low values of these parameters suggest good predictive capability and robustness against any overfitting.

To evaluate the reproducibility of the developed models, the validation set was reanalyzed in an independent different laboratory. This strategy simulates real-world applications where the predictive model may be applied across different labs or quality control environments using various spectroscopic setups. The use of these external datasets enables the evaluation of instrumental transferability, minimizing instrumental bias, and ensuring model portability. The same measurement procedures were followed during the analysis and the spectra were fully acquired within the predefined wavelength range and processed using the established chemometric models.

#### Novel variable selection strategy for chemometric optimization (GA-ICOMP-PLS)

A novel variable selection strategy named Genetic Algorithm with Information Complexity for Partial Least Squares (GA-ICOMP-PLS) was developed and implemented during the current study. This method integrates a standard binary coded genetic algorithm with an information-theoretic fitness function to optimize the selection of spectral variables used in PLS regression. In each generation of the genetic algorithm, a binary chromosome representing the presence (1) or absence (0) of spectral variables was evaluated. For each chromosome, a PLS model was constructed using only the selected wavelengths. Instead of using traditional RMSE as the sole criterion, the information complexity metric (ICOMP) was used to evaluate model performance. The ICOMP penalizes models that are overly complex or collinear by incorporating both log-likelihood and covariance structure complexity as follows; ICOMP = − 2 log L + 2 C(Σ) where L is the likelihood and C(Σ) is a complexity penalty derived from the covariance matrix of the residuals. This formulation ensures that selected variable subsets yield not only accurate but also parsimonious and stable models. The genetic algorithm was run for 50 generations with a population size of 30. Uniform crossover and bit-flip mutation (at a 1% rate) were applied. The solution with the lowest ICOMP score was selected as the optimal variable subset. This method was compared against GA-PLS and conventional full-spectrum PLS models to assess its effectiveness in variable reduction and prediction robustness.

#### Analysis of paxlovid^®^ and spiked human plasma

The recommended dosage of Paxlovid^®^ consists of one tablet containing 100.0 mg of RNV and two tablets containing 150.0 mg each of NMV. For sample preparation, RNV and NMV tablets were accurately weighed and grounded together. A portion equivalent to 100.0 mg of RNV and 300.0 mg of NMV was taken, sonicated in ethanol for adequate extraction, and then filtered through a 0.45 μm membrane filter. A final concentration of 1.0 mg mL^− 1^ RNV and 3.0 mg mL^− 1^ NMV was obtained upon adjusting the volume. Subsequent dilutions were made to achieve concentrations falling within the chemometric calibration range. The UV spectra of the prepared solutions were obtained at a measuring range of 190 – 400 nm and analyzed using the constructed models. The precision of the models was evaluated by calculating the relative standard deviation (% RSD) from five replicate measurements.

Human plasma was attained from the laboratories of October 6th University Hospital. Different spiked human plasma samples were prepared in centrifuge tubes by adding to them various aliquots of different concentrations of RNV and NMV with a certain volume of acetonitrile. The mixtures were vortexed for 1 min and then centrifuged at 3000 rpm for 30 min. The supernatants were collected and evaporated to dryness. Then the residues were dissolved in a fixed volume of ethanol. Afterward, acetate buffer (pH 4.0) was added to each sample, and the volumes were completed using ethanol and analyzed as previously described at the wavelength range of 190 – 400 nm using the constructed models.

#### Models comparative study

A comprehensive comparison was held between the models to evaluate their respective strengths and weaknesses. Different metrics such as RMSEC, RMSEP, % RE, % RSD, and r were employed during the study to address the models’ performance and applicability.

## Results and discussion

Ensuring uniform data sampling is essential for obtaining thorough and representative datasets, especially in multidimensional variable spaces. Our objective is to evaluate the impact of LHS on the predictive performance of multivariate models applied to spectrophotometric data of RNV and NMV. By ensuring stratified and space-filling sample generation, LHS is expected to yield more representative validation datasets. This technique ensures uniform and stratified coverage of the experimental space and is hypothesized to improve model performance by enhancing data diversity and space-filling properties. The MC technique was utilized to validate and evaluate the effectiveness of LHS. Various signal preprocessing techniques such as the Savitzky-Golay filter, spectral derivatization, and SNV smoothing have been incorporated to assess PLS model accuracy. This investigation aims to fill a methodological gap by coupling systematic sampling design with chemometric processing, thus offering a new dimension to model optimization in spectroscopic analysis.

**Fig. 2:**
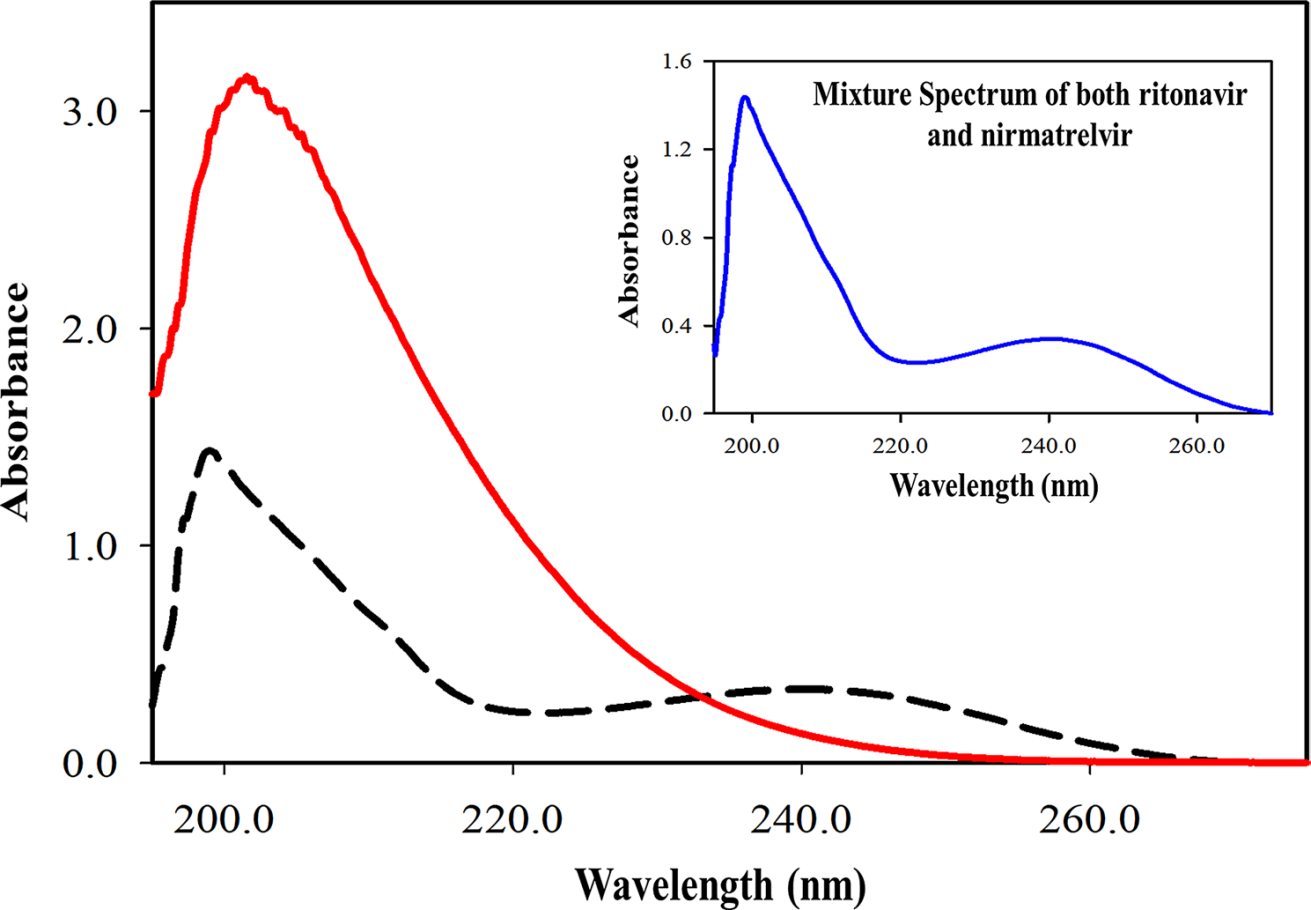
Zero order absorption spectra of 30.0 µg mL^− 1^ ritonavir (………) and 100.0 µg mL^−1^ nirmatrelvir (………) using ethanol as blank. The inset picture represents a **Mixture Spectrum** of the two components (25.0 µg mL^− 1^ ritonavir mixed with 5.0 µg mL^− 1^ nirmatrelvir)

Upon examining the UV spectra of RNV and NMV, a significant overlap was noticed (Fig. 2), making their interpretation more challenging. Thus, employing chemometrics with the LHS technique will be a reliable strategy for deciphering complex data and accurately predicting analyte concentrations through multifactor predictive analysis.

### Acquisition of absorption spectra

Adjusting procedure conditions is necessary for achieving accurate and consistent data. The UV spectral scans were performed in the 190–400 nm range to identify regions with substantial absorption features. A fast scan speed was chosen to record data at 0.1 nm intervals across the selected wavelength range, increasing the strength of the analysis while sustaining an equilibrium resolution. An ideal wavelength range of 200–260 nm (301 points) was utilized for the models’ construction. Wavelengths below 200 nm and above 260 nm caused model disturbances, however, medication spectra revealed considerable absorption below 260 nm, thus, wavelengths other than these ranges (200–260 nm) were excluded. A 0.2 nm range was chosen for data acquisition and models’ construction, ensuring sufficient resolution for capturing all spectral features required for accurate determination.

### Solvent selection and UV transparency consideration

Various solvents including water, methanol, ethanol, and acetonitrile were evaluated to identify the most suitable medium for the UV spectrophotometric analysis. Among these, ethanol and acetonitrile proved to be more suitable for our analytical purposes whereas water and methanol resulted in lower absorption intensities and reduced sensitivity. During the current study, ethanol was chosen as a greener alternative to acetonitrile, in alignment with the principles of green analytical chemistry. This choice prioritizes environmental sustainability, reduced toxicity, and operator safety. Although, acetonitrile is known for its higher UV transparency, particularly below 210 nm, preliminary spectral evaluations demonstrated that ethanol provided sufficient clarity and transparency in the 200–260 nm range. Well-resolved and quantifiable absorption bands of RNV and NMV were obtained with ethanol, without significant baseline drift or peak distortion. The success of the applied chemometric models, which yielded high prediction accuracy and minimal error metrics, supports the adequacy of using ethanol for this application. The trade-off in spectral transparency was carefully considered and ultimately deemed acceptable in favor of improved sustainability.

### Models construction

#### Signal preprocessing and principle component (PCA) enhancements

Before tailoring regression models, signal preprocessing and PCA tools were used to enhance and detect the variance structure within the dataset while searching for patterns or anomalies. Various preprocessing techniques were utilized during the current study. The PLS model was constructed twice, once using the raw absorbance data and the other using preprocessed signals to evaluate the effect of signal enhancement on model performance. Both models were built using the calibration and validation datasets. Combined sequences of the Savitzky-Golay filter, spectral derivatization particularly first derivative, and SNV smoothing were performed then, the refined spectra were later used to develop a PLS regression model for assaying RNV and NMV. The predictive power of these preprocessing steps was systematically evaluated using prediction plots for both drugs and then compared to the raw, original, unprocessed spectral data (Supp. Figure S1). Each technique serves a specific function in data optimization. Savitzky-Golay filter reduces random noise while preserving the shape of the spectral peaks while the derivatization process enhances the resolution between the overlapping peaks especially in complex mixtures and the SNV normalization procedure corrects the baseline shifts and scattering effects. Using the first derivative with other approaches provided superior predictive accuracy for RNV and NMV, contrary to the second derivative. The concentrations showed tighter clustering around the regression line with low values of RMSEC of 0.19 for both RNV and NMV, indicating minimal scattered residuals and higher model performance. However, despite the good performance of the second derivative, it exhibited a slight deviation from the regression line (Supp. Figure S1c) with RMSEC values of 0.26 and 0.27 for RNV and NMV respectively. This may be attributed to its higher sensitivity to noise that sometimes can affect model stability, if not properly optimized. The obtained results highlight the significance of using first derivative preprocessing, which balances noise reduction and spectral enhancement effectively, making it a preferable choice for this analytical model. This comparison demonstrated that the preprocessed data yielded better-defined features and improved model calibration, as visually evident in the preprocessing output plot. The spectral profiles after preprocessing were smoother and more resolved, indicating successful noise reduction and enhanced interpretability.

Supplementary Figure S2 demonstrates the PCA result, displaying both calibration and validation mixtures distributed along the first two principal components (PC1 and PC2) which are relative to the most significant spectral data variance. The PCA score plot indicated a well-structured sample distribution, with no clusters indicating outliers or abnormal grouping. Both sets, calibration and validation sets, aligned well within the same PCA space which illustrates the homogeneity of mixtures, also proving that validation samples are scoped within the calibration sample’s modeled space. This plot offered several advantages for the current study. Firstly, it verified dataset integrity in terms of systematic errors, giving additional confidence in the reliability of the models. Secondly, it supported the representativeness of the calibration set with respect to the validation set that is required for constructing predictive models. The PC1 reported a 99.46% of the total variance in the data, while PC2 explained only 0.45%. This reveals that PC1 alone captures nearly all the relevant information and variation within the dataset, making it the dominant axis for differentiating sample distribution. Although such a high percentage of PC1 may suggest variables’ redundancy or potential overfitting, this was deemed appropriate in our study as it reflects the strong co-linearity and consistent variation in the spectral dataset, especially after signal preprocessing procedures. These procedures are known to enhance spectral features, reduce irrelevant variation, remove baseline deviations, standardize analyzed data, and improve signal fidelity. Therefore, the high PC1 variance likely reflects meaningful chemical information, not noise. Additionally, the minimal contribution of PC2 suggests that most of the variability is aligned along a single direction, which confirms the robustness and consistency of the experimental design and sample selection. The color coding provided additional evidence to support the idea that the predominant source of variation aligns with concentration shifts. This is further supported by the excellent performance of the developed models, confirming that no overfitting occurred and that the dataset was both compact and information-rich.

#### Development and optimization of the chemometric models

Brereton et al. [54] multi-level multifactorial design was followed to prepare twenty-five mixtures as a training set (Supp. Table S1). Calibration values ranged from 5.0 to 25.0 µg mL^− 1^ for both RNV and NMV were prepared to ensure an even representation of concentration levels and analyzed using the developed models (Supp. Figure S3). Using solvent spectra as negative controls during the implementation of Brereton et al.’s multifactorial design is critical to ensure and strengthen signal specificity claims. In the current study, solvent-only spectra (blank) were checked to ensure spectral specificity. These solvent blanks, containing only ethanol as the diluent, displayed no measurable absorbance in the relevant analytical window (200–260 nm), thereby confirming the absence of interfering signals from the matrix. Besides and before recording the UV spectra of the analytes, a baseline correction was performed using the pure solvent (ethanol) to nullify its absorbance contribution. This ensured that subsequent spectral measurements reflected only the absorbance of RNV and NMV, thereby enhancing the specificity and reliability of the analytical signals. However, despite this supported conclusion of recorded signals stem primarily from the analytes of interest, future work may properly incorporate these blank spectra into the multivariate model calibration as negative control spectra to further validate specificity.

**Fig. 3:**
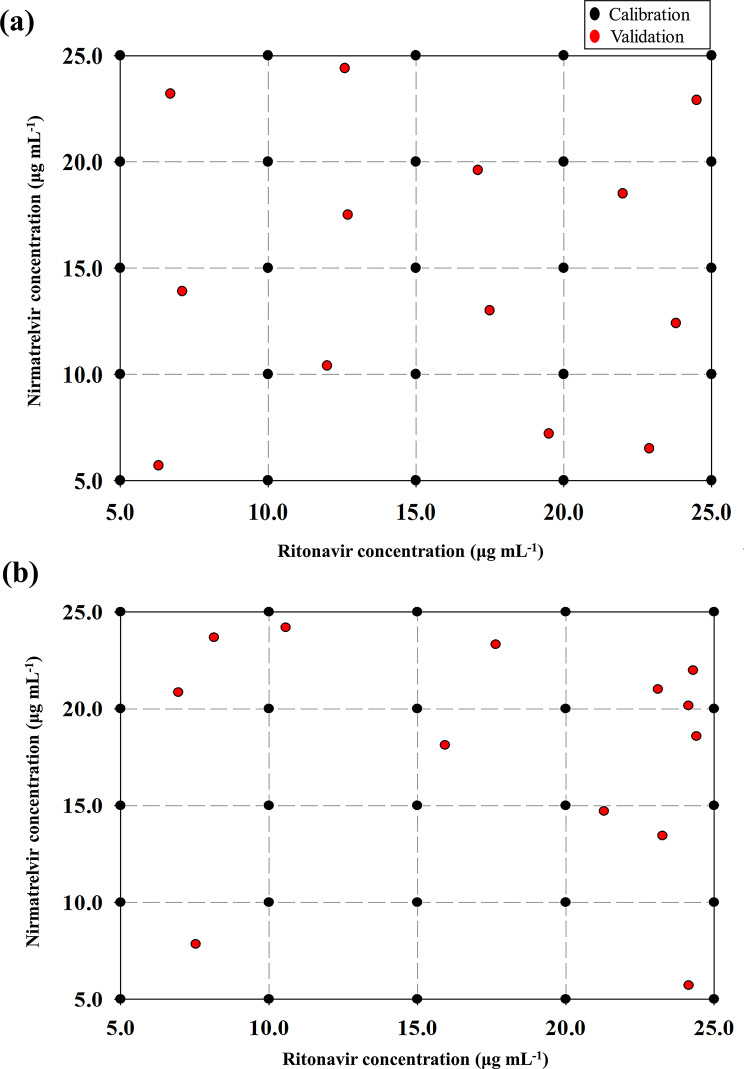
2D space-filling scattered plot indicating the validation set that was designed using (**a**) Latin hypercube sampling and (**b**) Monte Carlo random sampling

The validation set was designed to contain different mixtures with well-distributed and random concentrations using the LHS technique (Supp. Table S1). The concentrations were divided into 13 equal probability strata based on variable numbers and modeled mixtures (Fig. 3a) and then analyzed using the same procedures of the calibration set.

##### The rationale behind the validation set

In the current study, the implemented design strategies ensured that the model training and validation phases were supported by a comprehensive and balanced dataset. The total dataset consisted of 38 experimental mixtures and was partitioned into 25 calibration (approximately 65.8%) and 13 validation (approximately 34.2%) samples. This split adheres to the commonly used 2:1 ratio recommended in chemometric modeling to balance the amount of data used for model training versus independent evaluation as in the works of Brereton et al., who emphasize that such a ratio maintains a balance between model learning capacity and independent evaluation, especially when the total dataset is moderately sized. The selection of 13 points corresponds directly to the number of validation samples where each validation sample was drawn from a distinct stratum across the analyte concentration range, thereby reducing selection bias and maximizing diversity within the validation subset. This strategy avoids redundancy and enhances the reliability of performance metrics. Figure 3a demonstrates how the 13 validation samples selected via LHS are well-distributed across the RNV and NMV concentration space. Unlike random sampling (Fig. 3b), LHS ensures that the validation samples systematically span the entire range of the calibration space, reducing sampling bias and improving representativeness. This supports the justification for using 13 samples as they efficiently cover the design space without redundancy or clustering, even with a relatively small set size.

##### The PLS modeling and optimization

PLS expands the relation between response variables (component concentrations) and predictive factors (spectral data), making it a valuable tool in quantitative analysis for extracting profound data from non-selected datasets. When using a PLS model, the matrix of the spectral data is augmented with LVs to capture essential spatial lengths. Five LVs were identified as optimal for assessing RNV and NMV (Supp. Figure S4), resulting in minimal RMSEC values as shown in Table 1.

**Table 1:** Calibration set performance metrics for each model

Parameter		Model				
		PLS	GA-PLS	ANN	MCR-ALS	
**Ritonavir**	**Concentration range (µg mL** ^**− 1**^ **)**	5.00–25.00				
	**Slope**	1.0022	1.0001	0.9968	1.0000	
	**Intercept**	− 0.0655	− 0.0410	− 0.0212	1.2427 × 10^− 15^	
	**Correlation coefficient (r)** ^**a**^	0.9996	0.9992	0.9996	0.9993	
	**RMSEC** ^**b**^	0.21	0.19	0.19	0.10	
**Nirmatrelvir**	**Concentration range (µg mL** ^**− 1**^ **)**	5.00–25.00				
	**Slope**	1.0011	0.9971	0.9971	1.0000	
	**Intercept**	− 0.0257	0.0260	− 0.0210	− 2.2543 × 10^− 16^	
	**Correlation coefficient (r)** ^**a**^	0.9996	0.9997	0.9996	0.9992	
	**RMSEC** ^**b**^	0.21	0.16	0.19	0.11	

^a^ Data of the straight line plotted between predicted concentrations of each component versus actual concentrations of the calibration set

^b^ Root Mean Square Error of Calibration

##### The GA-PLS modeling and optimization

The predictive power of the PLS model was further enhanced using the GA tool. The latter explored various LVs within the constructed model by operating on different populations using several mechanisms including crossover and mutation to iteratively identify the most informative spectral regions. Different GA parameters were optimally configured as shown in Supp. Table S2, enables the exclusion of redundant wavelengths and the selection of key variables, consequently reducing the input data by approximately 36% and later used as inputs for the PLS model. Interestingly, the optimal number of LVs remained consistent at five, as also determined in the original PLS model (Supp. Figure S4), and the resulting RMSEC values were lower than those of the PLS model (Table 1), indicating better model accuracy with reduced data dimensionality. Although GA is capable of simultaneously optimizing both variable selection and LVs count, the consistent number of LVs is scientifically justifiable due to the highly collinear nature of the spectral data, where a certain number of LVs captures most of the variance. Increasing or decreasing LVs to a certain extent may yield no significant fitness advantage in the RMSECV function, and may introduce overfitting. In the current study, a constant LVs number was deemed appropriate as the number of LVs was initially optimized via the full-spectrum PLS using cross-validation, in addition, the GA was applied specifically for variable (wavelength) selection, not for optimizing the number of LVs. Therefore, this approach was adopted to reduce the search space and improve algorithm convergence, which is a widely accepted practice in GA-PLS modeling. Besides being unable for the GA’s fitness function (RMSECV) to suggest any benefit from altering the number of LVs indicates that five LVs already captured the essential variance while minimizing overfitting risks. This behavior reflects a convergence to the most tight and stable model configuration.

To address the susceptibility of the GA to local optimization, we introduced two enhancements to the GA-PLS model. First, a penalty term was added to the GA-PLS fitness function to discourage the over-selection of variables, promoting model sparsity and preventing overfitting. The regularization coefficient (λ = 0.05) was chosen empirically to balance predictive accuracy with model simplicity. Multiple GA runs with varied random sets and retained models were conducted to obtain the lowest cross-validated RMSE. These enhancements resulted in more robust wavelength selection (16 and 15 variables for RNV and NMV respectively) and improved prediction accuracy, as demonstrated by optimal RMSE values of 0.18 and 0.20 for RNV and NMV, respectively. The GA-PLS optimization strategy was visualized where a fitness evolution across 100 generations was plotted, highlighting convergence stability (Supp. Figure S5). The feature reduction process was monitored to assess the number of retained spectral windows over time. The decrease in fitness values as the window count stabilizes suggests optimal feature selection, thus reducing redundancy (Supp. Figure S5a). The fitness evolution plot highlights the effectiveness of GA modifications in avoiding local optima, a common issue in evolutionary algorithms, and ensuring a more stable convergence (Supp. Figure S5b). It demonstrates the progressive improvement in model performance, with a distinct separation between average and best fitness values. The increasing gap between best and average fitness values across generations suggests an improved balance between exploration and exploitation, ensuring robust spectral feature selection. However, the gradual decrease in spectral windows’ average number aligns with enhanced model efficiency, avoiding overfitting and demonstrating that redundant variables were successfully excluded without compromising predictive strength while preserving critical wavelengths that enhance model predictability (Supp. Figure S5c). This directly impacts spectral resolution, allowing for better differentiation among sample compositions. In Supp. Figure S5d, windows are utilized in up to 50 models, while others are rarely or never selected. These selected windows provide meaningful insights into chemically significant regions, confirming that GA optimization enhanced feature selection relevance. These findings contribute to improved model generalization and reduced bias.

##### The ANN modeling and optimization

**Fig. 4:**
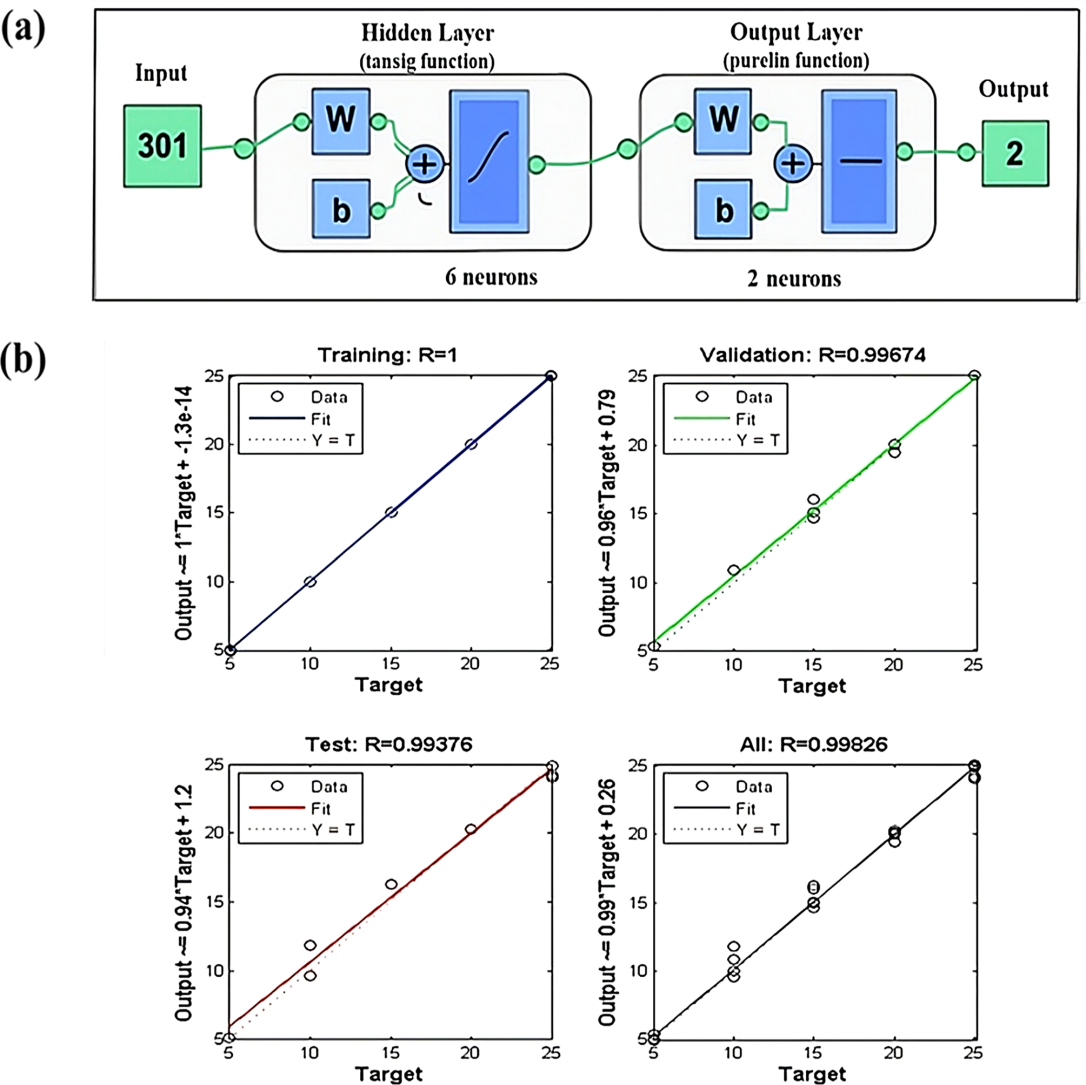
(**a**) Architecture of the ANN network for concentration prediction using different layers and (**b**) prediction plots of the training, validation, and testing sets using the ANN network

The ANN operates through an interconnected structure of different neurons where input data are received, processed, and transmitted (propagated) across the neuronal layers to generate output predictions. The desired network was built via the Levenberg-Marquardt algorithm (trainlm) where the iteration was repeated continuously through backpropagation for adequate network training [55]. A sigmoid (tansig) and Purelin-Purelin activation functions were used in the hidden and output layers respectively. The ANN architecture was designed based on empirical optimization and best practices to balance model complexity with predictive accuracy while avoiding overfitting. The constructed network demonstrated many layers for expecting the concentrations of the two analytes (Fig. 4a). The input layer included 301 neurons, corresponding to the number of selected spectral variables (wavelengths) retained after preprocessing. The output layer comprised two neurons, representing the concentrations of RNV and NMV. Through iterative experimentation using trial-and-error and cross-validation, six hidden neurons were selected as the optimal configuration, preventing overfitting while preserving generalization ability. This decision was based on a series of trials comparing network performance across hidden neuron counts ranging from 2 to 10. Using a larger number of hidden neurons may increase the risk of overfitting, especially given the limited number of training samples while fewer neurons led to underfitting and reduced model performance. Consequently, model performance across different numbers of hidden neurons was evaluated during the training and testing phases using metrics such as RMSE and r. The configuration with six hidden neurons yielded the lowest MSE (0.30) and highest r (0.9996) during validation, demonstrating superior predictive accuracy and robustness. The network was trained over 500 epochs, which provided sufficient convergence while maintaining computational efficiency. The dataset consisted of 25 calibration samples and was randomly partitioned into 70% training (18 samples), 15% validation (4 samples), and 15% testing (3 samples) using a fixed random subset to ensure reproducibility and model robustness while preventing overtraining. This partitioning strategy is widely adopted in ANN modeling to achieve a balance between model learning and generalization. Regression plots (Fig. 4b) confirm that the ANN achieved high predictive performance, with r values approaching unity across all data partitions. The model’s accuracy and adequacy were comparable to or surpassed other chemometric approaches used in this study such as PLS and GA-PLS, validating the chosen ANN configuration for this application.

##### The MCR-ALS modeling and optimization

**Fig. 5:**
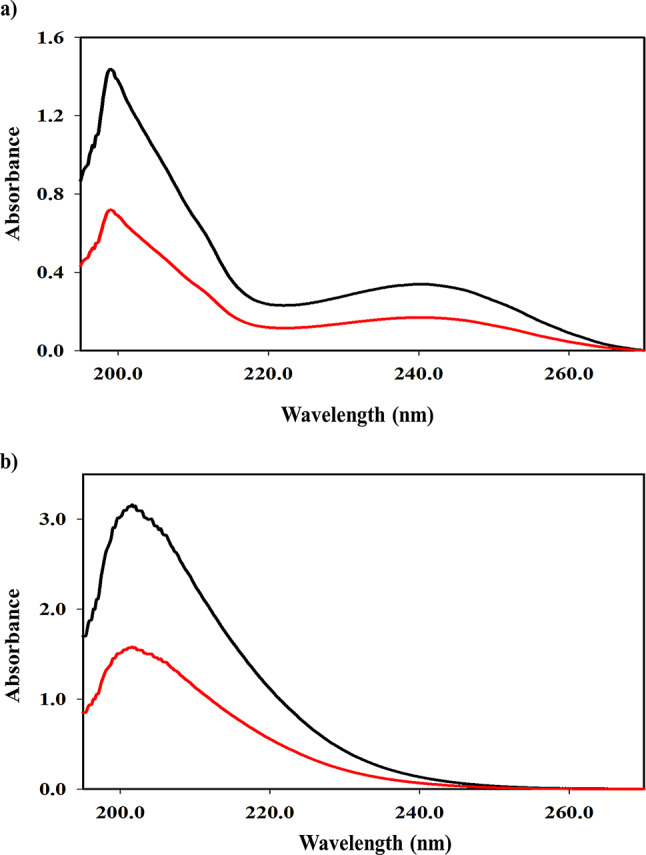
Original () and estimated spectra () of (**a**) ritonavir and (**b**) nirmatrelvir using MCR-ALS model

In the MCR-ALS model, the data were divided using the MCR bilinear pattern and then optimized using the ALS algorithms where non-negative constraints were employed on analyte concentrations and spectral data, ensuring that the confined values remain greater than or equal to zero [56]. The MCR-ALS algorithm was executed with a convergence criterion based on minimal change in residuals between iterations. Convergence was achieved after a sufficient number of iterations, with a residual standard deviation of 0.0289 and a fitting error of 5.91%, explaining 99.65% of the variance. These metrics confirm the stability and adequacy of the resolved concentration and spectral profiles. The low residuals and high variance explanation emphasize the reliability and stability of the resolved concentration and spectral profiles, validating the adequacy of the chosen model parameters. The MCR-ALS model was later used to assess the spectral criteria of RNV and NMV, indicating exceptional agreement between the calculated and original spectra for each element, as shown in Fig. 5. The convergence threshold used in the optimization was set to ensure less than 1% change in the residual error, consistent with typical practices in chemometric modeling.

##### Variable selection using GA-ICOMP-PLS

In the current study, a new hybrid variable selection method, GA-ICOMP-PLS, was developed to assess and enhance the robustness and parsimony of UV-based multivariate calibration models. This tool integrates GA with ICOMP as an information criterion, which balances model fit and structural complexity. The GA-ICOMP-PLS algorithm was implemented to select optimal spectral variables. It introduces a complexity penalty term based on the covariance structure of the prediction error. This penalizes overfitting and redundancy in variable selection, especially in collinear and high-dimensional spectral datasets. Model performance was assessed using leave-one-out cross-validation. The selected variables yielded prediction error values of 0.15 and 0.14, and r values of 0.9997 and 0.9998 for RNV and NMV, respectively, indicating strong predictive power and generalization. The model outperformed the standard GA-PLS in terms of generalizability and variable sparsity, as it consistently selected fewer but more informative wavelengths while maintaining or improving prediction accuracy. Compared to ANN, which relies heavily on architecture tuning and is prone to overfitting in small datasets, GA-ICOMP-PLS offered a more interpretable and reproducible alternative. While MCR-ALS excelled in spectral resolution, it did not provide variable importance insight or reduce the predictor space, both of which are critical for model simplification. Therefore, the GA-ICOMP-PLS strategy serves as a clever, efficient, and interpretable variable selection tool, aligning with the study’s green-and-white analytical goals. Its integration represents both a methodological innovation and a practical contribution to chemometric modeling, particularly in pharmaceutical analysis where spectral overlap and data redundancy are common.

#### Analysis of calibration and validation sets

The developed models were employed to determine the components’ concentrations. The % R, % RE, % RSD, and RMSEP were evaluated. The reduced values of RMSEP and % RSD in the validation set indicate that the models have high predictability and precision (Supp. Table S3). To ensure the models’ accuracy, a comprehensive analysis of the dosage form and human plasma is performed and five average determinations are used to assess the model’s precision. The models’ reproducibility was further assessed by analyzing the external datasets in a different laboratory. Precise results (% RSD) and low RMSEP values were obtained, especially by ANN and MCR-ALS models (Supp. Figure S6).

**Fig. 6:**
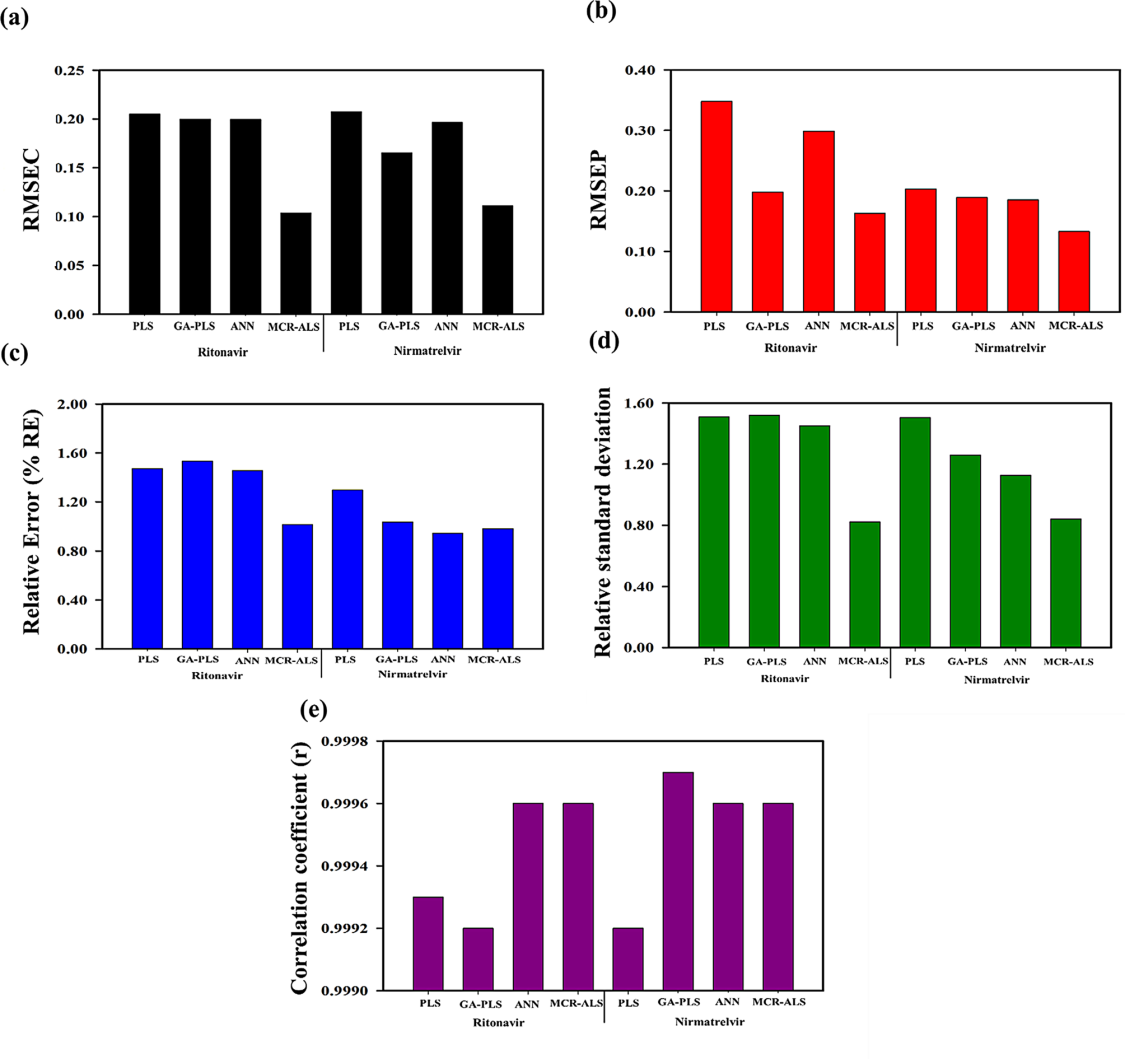
(**a**) The RMSEC values of the studied components calculated using the calibration models, (**b**) The RMSEP, (**c**) the relative errors, (**d**) the relative standard deviation, and (**e**) correlation coefficient values calculated using the validation set

The column chart of Fig. 6 shows the RMSEC, RMSEP, % RE, % RSD, and r calculations for RNV and NMV in both series, calibration, and validation. Significant findings have been obtained for each component where the error values for both sets have been reduced with higher correlations between the actual and predicted data, suggesting the models’ efficiency and reliability for evaluating complex mixtures. Results indicated that LHS offered superior data distribution and enhanced the performance of all chemometric models. Despite the affirmative results of the constructed models, the ANN and the MCR-ALS models were established as effective models since they produced lower error values of RMSEC and RMSEP compared to the former models. To accurately compare the predictive performance of these models, RMSEP values were computed and benchmarked against the PLS model. The MCR-ALS model yielded 53.1% and 34.6% relative reductions in the RMSEP for RNV and NMV, respectively compared to the PLS, while the ANN model achieved 14.1% and 8.9% relative improvements for RNV and NMV, respectively. These values represent relative enhancements in predictive accuracy, calculated as the percentage reduction in RMSEP compared to the PLS baseline. This distinction highlights the improved generalization of the advanced models, particularly MCR-ALS, and supports their effectiveness in capturing the underlying variance in the spectral data.

Notably, the ANN model achieved a slightly lower RMSEC value for RNV (0.19) compared to that of the PLS model (0.21), indicating a 2.7% numerical improvement. However, a statistical analysis using a paired t-test was conducted to evaluate the statistical significance of this difference, yielding a p-value above the conventional threshold of 0.05. This result suggests that the difference in RMSEC between the two models is not statistically significant, indicating that both models exhibit comparable predictive accuracy. Given this closeness in performance, ANN still exhibited marginally better RMSEC and RMSEP, indicating its practical superiority in the current dataset. These findings support the complementary strength of both approaches. Consequently, to provide a more holistic model evaluation, additional performance metrics such as % RE, % RSD, and r were also considered, as these offer complementary insights into model robustness, error magnitude, and predictive power. Strong linear relationships between predicted and actual concentrations, with r values of 0.9996 for both RNV and NMV have been demonstrated. The average % RE and % RSD values were below 2% for both drugs across all models, indicating high prediction accuracy. These findings suggest that the developed models provide reliable and robust quantification of the target analytes with minimal deviation from true values, confirming that the use of LHS improves calibration integrity, supports hypothesis validation, and enhances model reliability in the analysis of binary pharmaceutical mixtures.

These reductions are not only statistically significant but also practically meaningful for better model generalization. This improvement is critical in both clinical and industrial settings, where predictive accuracy directly influences decision-making. By minimizing the risk of underestimation or overestimation of drug concentrations, particularly in low-dose formulations, the models help ensure dose uniformity, patient safety, and compliance with regulatory standards. Additionally, increasing method precision may lead to fewer batch rejections and more consistent product release in quality control laboratories, leading to cost efficiency and more process reliability. Thus, a balanced interpretation of RMSEC, RMSEP, % RE, and r values provides ideal predictive accuracy for the developed models. This is particularly valuable for fixed-dose combination therapies where small deviations in drug content can lead to therapeutic inefficacy or toxicity.

### Sampling strategy comparison: LHS versus Monte Carlo

To further support sampling strategy evaluation, a comparative study between LHS and the Monte Carlo technique (MC) was performed. In the MC approach, the dataset was randomly split giving a validation set comprising 13 mixtures. Figure 3 demonstrates the distribution of validation samples selected by each technique across the experimental concentration space of RNV and NMV. In the LHS (Fig. 3a), validation samples are evenly distributed across the entire range of concentration levels for both analytes. This stratified coverage ensures that all regions of the experimental space are proportionally represented, which helps to enhance model robustness, minimize overfitting, and yield more reliable error estimates across unseen samples. In contrast, the MC sampling (Fig. 3b) reveals a random distribution that resulted in clustered validation points, particularly concentrated in the upper-right region (higher concentration values). Some areas, especially the lower-left quadrant, are entirely unsampled. This uneven distribution could lead to biased model evaluation and underrepresentation of edge cases, compromising the generalizability of the model. Performance metrics including RMSEP, % RE, and r were computed and compared to the PLS results. The MC approach yielded acceptable predictive performance, with RMSEP values of 0.43 and 0.41, % RE values of 1.841 and 1.946, and r of 0.9981 and 0.9979 for RNV and NMV respectively. Although both methods provided satisfactory results, the comparative analysis highlighted that LHS consistently delivered more stable and reliable performance. This is attributed to its stratified sampling strategy, which ensures more uniform coverage of the experimental space. In summary, LHS demonstrates a clear advantage in providing a more systematic and balanced validation set, which supports our choice of LHS as a preferred sampling method for chemometric model development and performance evaluation.

### Interpretation of sample composition, spectral behavior, and model accuracy

**Scheme 1:**
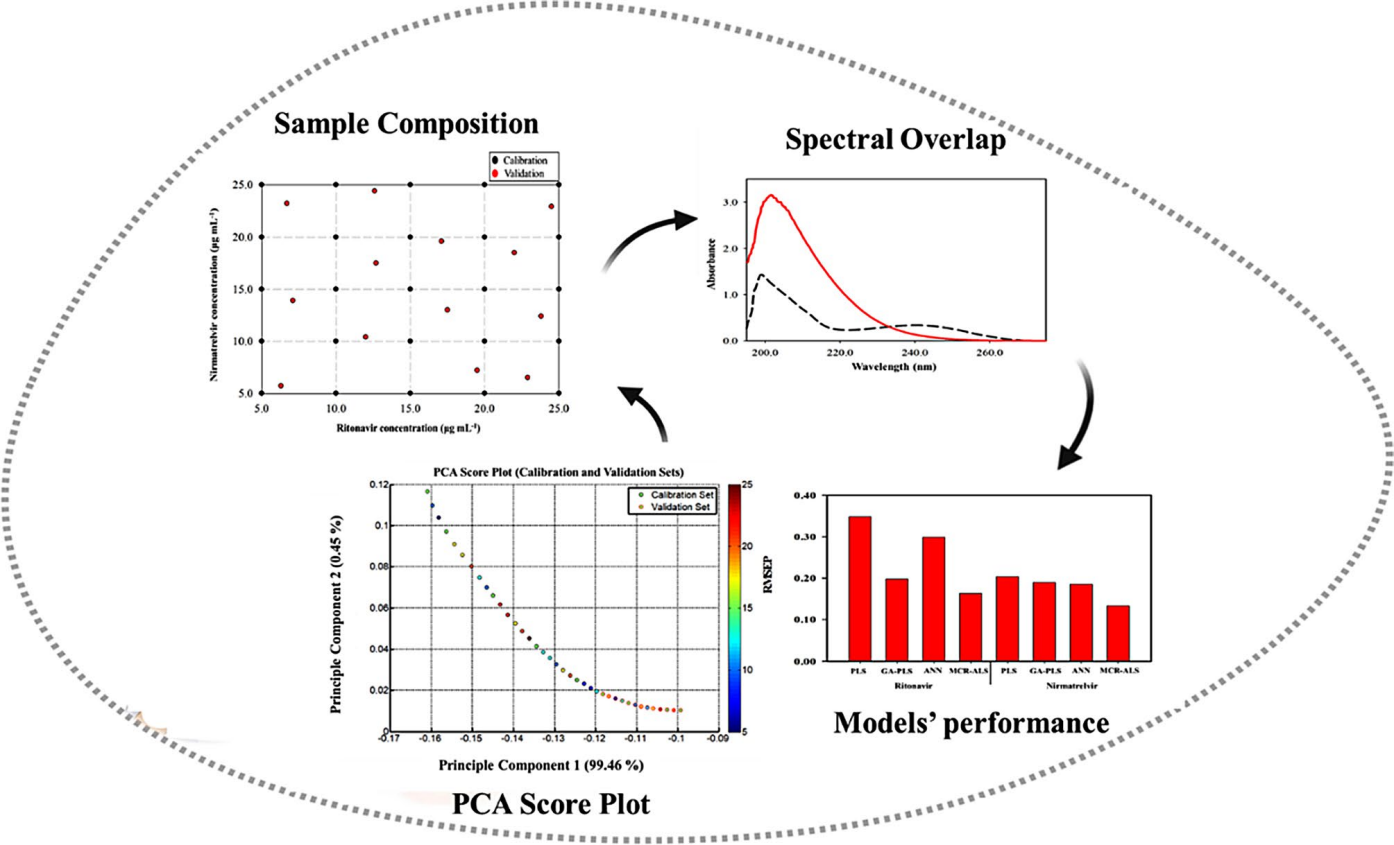
Relationship between sample composition, spectral overlap, and models’ performance

To provide a deeper insight into the modeling behavior, we investigated the underlying spectral and chemical properties of RNV and NMV. Integrating both methodological and sample-based insights helped the current study to develop models that are not only statistically robust but also chemically interpretable and generalizable to real-world applications. Both RNV and NMV exhibit considerable spectral overlap in closely located wavelength regions (between 200 and 260 nm) due to their aromatic and shared conjugated structures, posing a challenge for direct spectrophotometric resolution. Their lipophilicity and potential matrix interactions in biological samples further complicate their simultaneous quantification using traditional univariate analysis and necessitate the deployment of multivariate strategies not only for analytical separation but also to better understand and model the intrinsic physicochemical behavior of the two drugs. The success of GA-PLS, ANN, and MCR-ALS in minimizing drugs’ cross-interferences can be attributed to their ability to isolate subtle differences in the absorption features of the two compounds. GA-PLS enhances models’ interpretability through the selection of only the most informative wavelength variables, reducing noise and collinearity. ANN stands out in capturing nonlinear relationships within the spectral data, modeling complex interactions that conventional linear methods might miss. Its adaptive learning structure allows it to generalize well across diverse data distributions, making it particularly effective when trained on well-structured datasets. Meanwhile, MCR-ALS leverages bilinear decomposition and mathematical constraints to extract pure component spectra even in highly entangled regions. Additionally, the predictive enhancements observed with LHS can be explained by its ability to generate diverse mixture compositions that subject the model to a wider range of sample variation. This enhanced data diversity improves both spectral resolution and model generalization. These improvements were quantitatively supported by metrics such as higher r values, lower RMSEP, and reduced bias, while also being chemically justified based on the behavior of UV-active functional groups within the 200–260 nm region. Moreover, the hybrid GA-ICOMP-PLS method optimized the spectral variables used in PLS regression, attaining strong predictive power and generalization. The PCA was further used where score plots revealed improved dispersion and coverage of the chemical space, indicating reduced collinearity and better sample representativeness. Signal preprocessing techniques were employed to emphasize spectral differences and reduce noise, allowing for clearer identification of analyte-specific absorbance regions. These enhancements contributed to more meaningful wavelength selection in GA-PLS and improved interpretability of loading plots in PLS, demonstrating that robust sampling directly benefits model structure and interpretability. Therefore, the relationships among sample composition, spectral behavior, and model accuracy were not only statistically validated but also chemically and structurally rationalized (Scheme 1). These results support our hypothesis that using systematic LHS sampling techniques significantly enhances the robustness and predictive accuracy of multivariate models. The integration of LHS with conventional chemometric tools improves reliability and interpretability in the analysis of binary pharmaceutical mixtures.

### Chemometric models strategy

In the current study, creativity was implemented through the combination of experimental design and chemometric techniques tailored to UV spectrophotometric data. The chemometric models were employed within a carefully structured framework to maximize their predictive accuracy and robustness. A multilevel multifactorial design was followed for the generation of the calibration samples while LHS was used for the validation sample selection. A custom GA-PLS was utilized for variable selection, incorporating an RMSE-based fitness function and fixed random set to ensure repeatability and avoid convergence to local minima which is a common limitation of evolutionary algorithms. The ANN architecture was optimized by testing different hidden layer configurations and applying fixed partitioning sets for data consistency. In addition, signal preprocessing techniques (Savitzky-Golay filtering, spectral derivatization, and SNV correction) were applied to enhance the spectral signal-to-noise ratio and improve model input quality. These strategies, although based on established tools, reflect a thoughtful and integrated chemometric approach that enhances both predictive performance and experimental reliability. Furthermore, developing the GA-ICOMP-PLS method enhanced the robustness and parsimony of the UV-based multivariate calibration models.

### Analysis of paxlovid^®^ dosage form and spiked human plasma

We employed the suggested models to calculate the concentrations of RNV and NMV in Paxlovid^®^ tablets. The investigation showed great dependability at the claimed concentrations, meaning that medicinal excipients did not affect the results, giving acceptable accuracy with a low value of standard deviation (Table 2). Moreover, the applicability of the developed models was further assessed through the determination of RNV and NMV in various spiked human plasma samples. Despite the potential challenges posed by the plasma matrix, the results confirm the method’s selectivity and reliability in the presence of complex biological matrices, supporting its potential use in pharmacokinetic or bioequivalence studies. (Table 3).

**Table 2:** Quantitative assay of Ritonavir and nirmatrelvir in Paxlovid^®^ medication with statistical evaluation of the obtained results with a reported HPLC one

Parameters	PLS		GA-PLS		ANN		MCR-ALS		Reported method ^b^ [40]	
	RNV	NMV	RNV	NMV	RNV	NMV	RNV	NMV	RNV	NMV
**Mean R %** ^**a**^	98.58	100.64	99.60	100.81	98.17	99.07	99.69	99.57	99.45	100.19
**SD** ^**a**^	1.451	1.408	1.309	1.580	1.364	1.336	0.630	0.826	1.230	0.945
**Variance** ^**a**^	2.105	1.982	1.713	2.496	1.860	1.785	0.397	0.682	1.513	0.893
**n**	5								5	
**Student t-test** ^**c**^ **(2.306)**	1.023	0.593	0.187	1.519	1.558	1.530	0.388	1.105		
**F-test** ^**c**^ **(6.39)**	1.392	0.450	1.133	1.650	1.230	1.999	0.262	1.309		

^a^ Average of five determinations

^b^ Shimadzu reversed-phase HPLC system (Japan) equipped with an SPD-20 A UV-Vis detector, LC-20AD pump, and SIL-20 A auto-sampler was utilized during the study. A Prontosil C_18_ column (Germany) (150 mm x 4.6 mm, 5 μm particle size) maintained at 25 °C was used as the stationary phase. The mobile phase consisted of ethanol and water (80:20 v/v) and delivered at a flow rate of 1.0 mL min⁻¹. The samples were prepared in the same solvent system as the mobile phase to maintain consistency. The injection volume was 20 µL and detection was performed at 215.0 nm

^c^ The values in parentheses represent the tabulated two-tailed values at p = 0.05

^*^ RNV: ritonavir, NMV: nirmatrelvir

**Table 3:** Quantitative assay of Ritonavir and nirmatrelvir in spiked human plasma matrix using established chemometric models

	Drugs		Recovery (%) ± RSD ^a^		
		PLS	GA-PLS	ANN	MCR-ALS
**Spiked human plasma**	**RNV**	96.63 ± 2.251	97.20 ± 2.111	97.37 ± 1.938	98.68 ± 1.813
	**NMV**	97.05 ± 2.406	98.68 ± 2.133	97.29 ± 1.629	97.92 ± 1.677

^a^ Average of five determinations

^*^ RNV: ritonavir, NMV: nirmatrelvir

### Comparative statistical analysis of different methods

Using advanced analytical techniques such as LC-MS/MS for comparative analysis can significantly enhance the validation of analytical methods and boost confidence in their applicability. However, their high cost and limited availability often restrict their use in routine quality control, making them impractical for certain laboratories. Consequently, to assess the performance of the proposed chemometric method, the reported HPLC method [40] was conducted in our laboratory under the same experimental conditions using the same set of prepared samples. This allowed a direct comparison between the two methods in terms of accuracy and precision. The results obtained from both methods were statistically compared using Student’s t-test and F-test at a 95% confidence level to determine whether there were any significant differences in the mean values (accuracy) and variances (precision) of the results. The results are summarized in Table 2 where the calculated t and F values were found to be lower than the critical values, indicating that there is no significant difference between the two methods at the 95% confidence level. These findings confirm the suitability of the proposed method as a reliable, cost-effective, and practical alternative for routine analysis, especially in laboratories where access to advanced instrumentation such as LC-MS/MS is not feasible.

### Assessment of solvents’ greenness

**Fig. 7:**
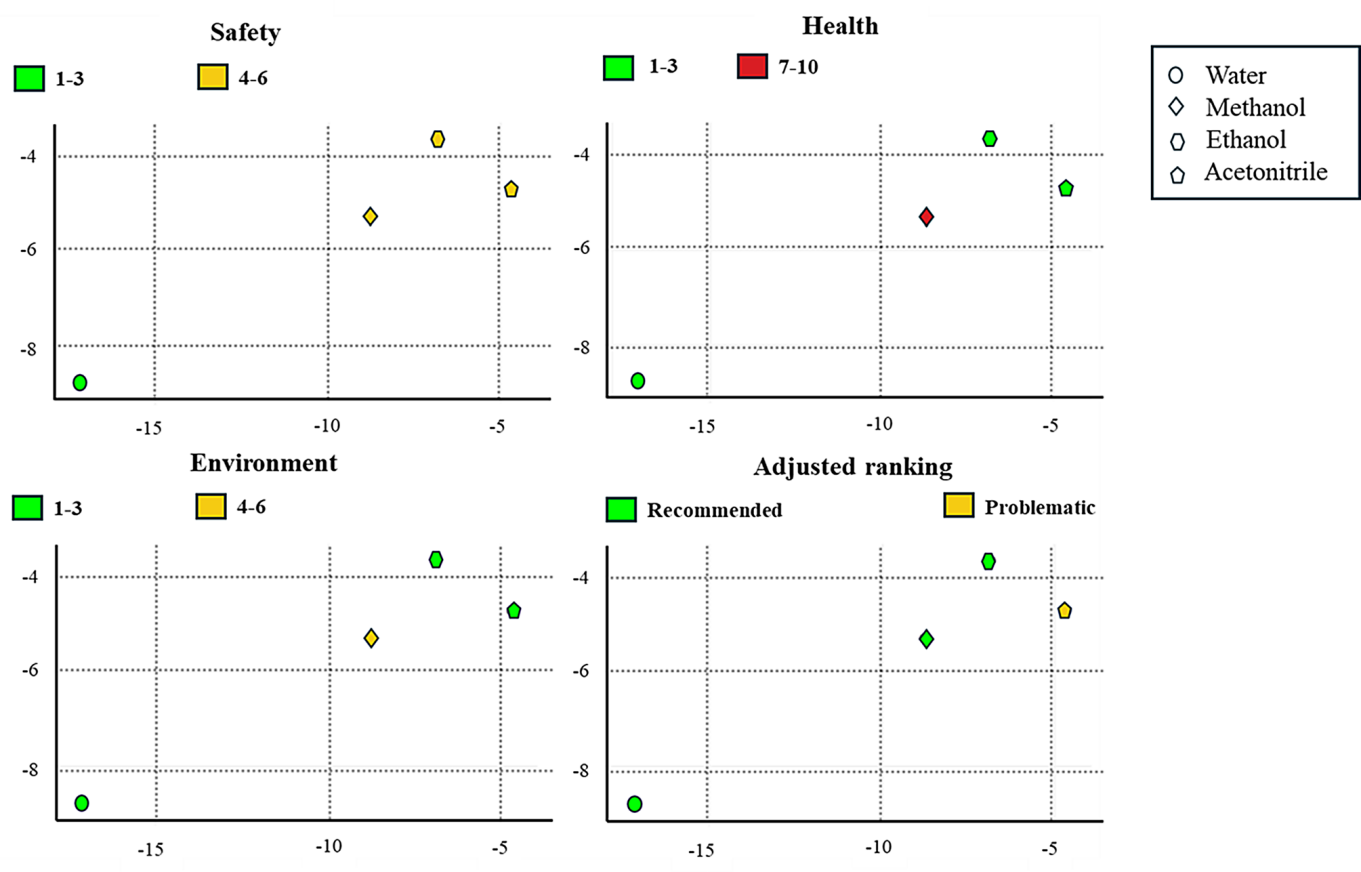
The SHE impacts of water, methanol, ethanol, and acetonitrile using the ACS GCIPR solvent selection tool

**Fig. 8:**
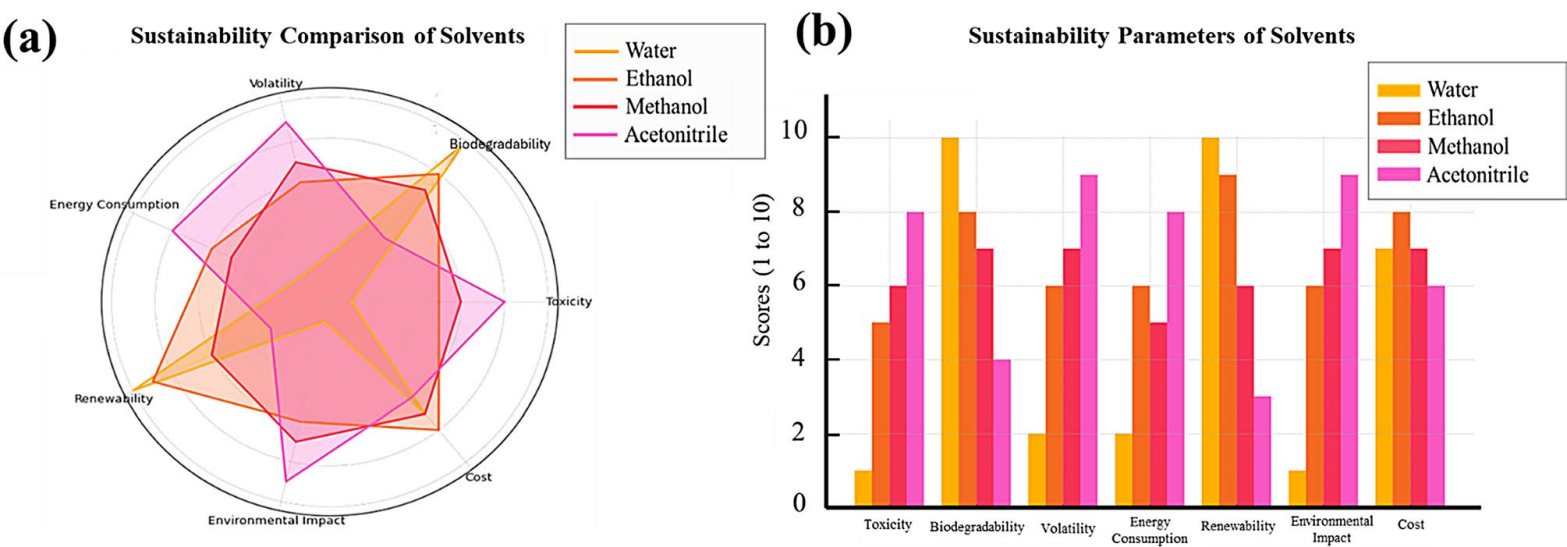
(**a**) Radar plot and (**b**) bar chart comparing the sustainability of solvents including water, methanol, ethanol, and acetonitrile using the SUSSOL tool

Proper selection of analytical solvents can significantly minimize the environmental footprint of different analytical processes. In this study, two evaluation tools such as “the American Chemical Society Green Chemistry Institute Pharmaceutical Roundtable solvent selection tool” (ACS GCIPR tool) and “the Sustainable Solvents Selection tool” (SUSSOL tool) were implemented and fully discussed in the Supplementary material file. These tools provide sufficient insights into solvent toxicity, biodegradability, energy efficiency, and ecological impact. Based on the SHE criteria of studied solvents, the ACS GCIPR tool was utilized to select the most appropriate solvent for RNV and NMV. Figure 7 highlights that water is the greenest option, followed by ethanol, methanol, and lastly, acetonitrile. Unfortunately, water was not suitable for conducting the current analysis due to the lack of solubility and sensitivity requirements. Consequently, ethanol was selected as the second-best choice, proving to be an effective solvent, producing accurate and sensitive results while aligning with sustainability goals. Moreover, the SUSSOL tool was used to assess the solvents studied, and the results were visualized using a radar plot (Fig. 8a) and bar chart (Fig. 8b). The charts confirmed the findings of the ACS GCIPR tool, indicating that water is the optimum choice followed by ethanol, methanol, and acetonitrile. These visualizations provide a comprehensive analysis by using solvents that align with both the analytical requirements and sustainability goals.

### Sustainability assessment of the developed models

**Fig. 9:**
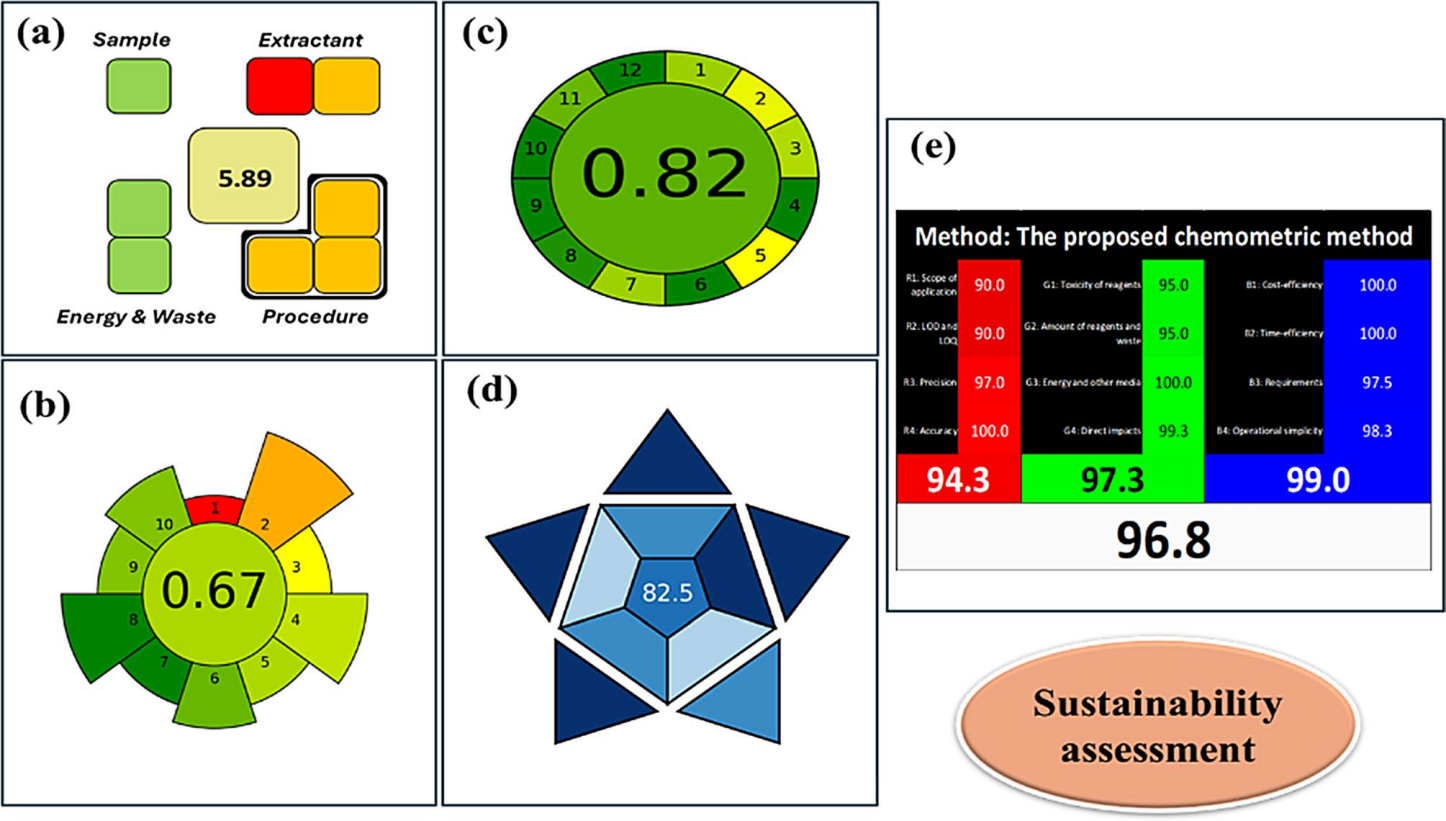
Sustainability assessment results of the developed method using (**a**) SPMS, (**b**) AGREEprep, (**c**) AGREE, (**d**) BAGI, and (**e**) RGB12 evaluation tools

Environmental contamination caused by hazardous solvents necessitates the development of ecological methods, using sustainable alternatives instead of noxious reagents, and reducing solvent consumption. In modern chemistry, several metrics; greenness, whiteness, and blueness are integrated to evaluate different analytical processes and develop more sustainable practices, protecting ecosystems and human health while maintaining the method’s efficiency and effectiveness. Greenness metrics mainly focus on the environmental friendliness of the method by referring to factors such as solvent toxicity, energy consumption, and waste generation. In contrast, whiteness is a much broader concept than the GAC that is introduced to assess the overall sustainability of the analytical method, integrating not only greenness concepts but also method performance and practical efficiency regarding accuracy, precision, and cost-effectiveness, reflecting the method’s “fitness for purpose”, thus offering a balanced overview of sustainability and technical reliability. On the other hand, blueness has been recently introduced through the RGB12 model, which highlights analytical efficacy and innovation, assessing the balance between robustness, safety, and environmental responsiveness. Despite all these diverse features, it is essential to thoroughly choose and apply appropriate metrics for the methods’ evaluation. The current study provided a full assessment report indicating the sustainability and wide applicability of the developed models. More than one assessment tool, SPMS, AGREE, AGREEprep, RGB12, and BAGI, were used to obtain synergistic results and reliable integrated information regarding the developed models to confirm their analytical utility and practicality (Fig. 9).

#### Greenness assessment

AGREEprep [57] and AGREE [58] along with the recently introduced metric, SPMS [59], were successfully applied for models’ evaluation. Introduced in 2023 by Gonzalez-Martín and colleagues, the SPMS serves as an explicit tool to assess the ecological sustainability of sample preparation. It focuses on assessing nine different parameters divided into four categories that directly affect the performance of most extraction methods and also compromise method sustainability. Each category is assigned a certain weight in the total score based on its influence on the method’s endurability. A clock diagram represents the results where each step appeared as a colored square ranging from good to bad. Additionally, AGREEprep was introduced, by Pena-Pereira and colleagues, as a new feature that is added to the AGREE tool and emphasizes the sample preparation step. It is considered the first metric that converges on sample preparation and depends on ten criteria covering different green aspects of sample preparation. Each criterion is visually represented with different colors and a numerical middle appearing score in round pictogram. The criterion color instantly alters after data inputs, helping in identifying the procedure’ weak and strong points. Meanwhile, AGREE offers a quantitative comprehensive assessment for method sustainability based on the 12 rules of GAC, including four pioneering parameters. A colored graph ranged from dark green for exceptional greenery to dark red for serious flaws. Both AGRRE and AGREEprep could be used as complementary tools to each other, where AGREEprep emphasizes sampling evaluation while AGREE provides a full greenness estimation. Significant scores of 5.89, 0.67, and 0.82 were successfully achieved using SPMS, AGREE, and AGREEprep metrics, respectively which indicated the higher alignment of the developed models with the GAC principles (Fig. 9).

#### Whiteness assessment

WAC integrates broader sets of criteria according to the GAC principles to provide a comprehensive evaluation of the analytical methodologies [60]. It connects the process’s environmental aspects with the method functionality and economic viability that appears in the colored model of RGB12. This recently developed tool scores analytical methods based on 12 criteria (principles) related to environmental impact, safety, and resource use, producing a single percentage score ranging from 0 (least green) to 100% (ideal whiteness). It provides a holistic assessment by integrating environmental (green), performance (red), and practicality (blue) attributes, collectively representing the whiteness of an analytical method. Each principle is considered during evaluation where the whiteness score indicates the worst and ultimate performance. During the present work, the criteria of WAC were pursued using the RGB12 model. A score of 96.8% was effectively attained by the established models which is indicative of their wide applicability, eco-friendliness, and effectiveness (Fig. 9). This excellent score stems from the use of ethanol as a greener solvent, the absence of toxic reagents such as acetonitrile, and the overall low energy and waste footprint of the method. Such an evaluation supports the method’s suitability not only from an analytical performance perspective but also from a sustainability and safety standpoint. Given its comprehensive structure and reproducibility, the RGB12 score offers a transparent and advanced evaluation framework, thus increasingly recognized in the GAC society as a reliable indicator of methodological sustainability [61].

#### Blueness assessment

A modern assessment tool named the BAGI [18] was freshly proposed to fully evaluate methods’ blueness and provide a thorough approach for measuring the practical disciplines of WAC. It is a conceptual framework that is used to assess the economic viability and overall applicability of different analytical methods. It completes the green measurements by evaluating ten key characteristics. Each attribute is graded using a sequential blue color scale from 1 (worst) to 10 (best). Discrete colors ranging from dark blue, blue, light blue, and white appear to represent the high, medium, low, and even lack of fulfillment of the developed method with the established criteria, respectively. BAGI’s simplicity and easy pertinency encourage several analysts to incorporate it within their assessment strategies. During the present study, the merits of BAGI were carefully pursued and an appropriate score of 82.5 was achieved (Fig. 9). The results obtained indicated the good practicality and utility of the developed models. This score reflects the use of ethanol which is a low-toxicity and biodegradable solvent along with the method’s low energy consumption, limited reagent volumes, and minimal waste generation. The BAGI score is particularly noteworthy when benchmarked against other methods. Conventional methods using hazardous solvents such as acetonitrile often yield BAGI scores between 55 and 70 largely due to their toxic criteria and higher waste burden. In contrast, methods following GAC principles tend to fall within the 80 – 90 range, similar to the current study. Thus, the BAGI score not only confirms the method’s green profile but also positions it favorably relative to traditional and recently optimized methods. Achieving a high score supports the method’s potential for routine use in environmentally conscious analytical laboratories.

### Merits of integrating multiple metrics

While greenness focuses on eco-friendliness, whiteness reflects a thorough view including method quality and applicability, and blueness highlights the innovation and analytical value of the method. Therefore, incorporating multiple assessment tools for both greenness and practicality evaluation provides a well-representative strategy able to underline the method’s sustainability and attainability. It offers a more nuanced, adaptive, and resilient framework for complementary and consistent analysis which ultimately enhances scientific, environmental, and economic outcomes. Currently, this study holds the idea of integrating multiple assessment tools such as SPMS, AGREE, AGREEprep, RGB12, and BAGI for complete perspectives, balanced decision-making choices, improved iteration, and mitigating risks by identifying blind spots. Each metric has its own specifications, advantages, and even certain limitations. So, it is necessary to carefully choose and apply the most appropriate metric according to the method’s intended desires.

For example, sample preparation is regarded as the key, unavoidable, least eco-friendly step during the entire analytical procedure, especially when dealing with complex samples. Using the SPMS tool offers a comprehensive and detailed assessment of the entire sample preparation stage, focusing on environmental sustainability and operational efficiency, while emphasizing resource optimization through the reduction of energy and chemical waste. Furthermore, AGREEprep is comparable to the SPMS tool, as both concentrate on sample preparation. However, AGREEprep places a greater emphasis on environmental safety and chemical hazards while maintaining a quantitative approach, thereby facilitating an effective comparison of various methods. Unfortunately, focusing exclusively on sample preparation, without considering the entire analytical method, provides limited insights for method evaluation. Using the AGREE framework offers comprehensive coverage and extensive assessments across various environmental factors relevant to the entire analytical method. It evaluates sample preparations, instrumentation, reagents, and energy consumption, rendering it more thorough than metrics focused solely on specific parameters. All three tools are complementary to each other since AGREE lacks specificity for sample preparation however, they all are unable to consider method practicality and economic viability which necessitates the incorporation of BAGI and RGB12 metrics. BAGI emphasizes method practicality and applicability for real-world applications, complementing greenness metrics through its simplicity and versatility, however, it cannot assess the direct environmental impacts which require the usage of additional tools such as the RGB12 algorithm to entirely evaluate the method’s efficacy. The RGB12 provides a more holistic view and balanced assessments by considering a wide range of sustainability factors, yet, the metric calculation complexity, multiple data inputs and unprioritized sample preparation or waste management offer certain limitations that hinder method applicability.

Ultimately, each tool has its strengths and limitations, and their choice depends on the specific aspect of sustainability or applicability being prioritized. Employing multiple tools helps in addressing all aspects of sustainability, from environmental impact to practical applicability, leading to more robust, responsible, and ethical scientific practices.

### Literature survey

In the current study, a sustainability study was performed to evaluate the greenness of the developed method and compare it with the reported ones (Supp. Figure S7). Different aspects such as fatal materials usage, energy utilization, and waste generation were assessed using the previously mentioned metrics. Regardless of the resemblance between the reported methods in their greenness, the established method was slightly capable of outdoing the published ones. Conversely, a resemblance was noted between the developed method and a reported spectrophotometric method [53]. Despite the greenness assessment employed by the reported method, the current study incorporated additional metrics such as the ACS GCIPR and SUSSOL tools to assess solvents’ sustainability. The ACS GCIPR tool was able to classify solvents into different categories according to their toxicity, flammability, and environmental impact. The tool categorized water and ethanol as ‘Recommended’ solvents based on their SHE profiles of being safe, healthy, and environmentally benign, while methanol and acetonitrile were less favorable. The SUSSOL tool further validated this selection as it provided a detailed comparison between solvents based on various sustainability parameters such as toxicity, biodegradability, volatility, and energy consumption. Using these advanced tools provides a more robust and detailed evaluation of solvent sustainability.

Additionally, the current study introduced a pioneering statistical LHS technique coupled with chemometrics models. This technique offered optimal and well-interpreted samples with comprehensive space coverage which enabled not only a robust evaluation of the models’ predictive powers but also reduced waste and resource consumption. Relying solely on conventional spectrophotometric techniques lacks the advanced optimization and validation capabilities offered by both chemometric modeling and LHS techniques. This emphasizes the method’s significant role of being a superior and more efficient sustainable alternative to reported methods, especially conventional spectrophotometric methods.

## Conclusion

Random sampling often offers barren limitations that weaken the quality and reliability of data analysis. During the present work, we hypothesized that implementing LHS with signal preprocessing techniques will significantly enhance the predictive performance and robustness of the developed models. Firstly, PCA was conducted to visualize the underlying structure and variance in the spectral data. The clear and consistent distribution of both sets in the PCA score plot indicates the absence of outliers and confirms the homogeneity of the dataset. The alignment of spectral variation to concentration indicates that the data is appropriate for multivariate calibration analysis. Accordingly, four chemometric models such as PLS, GA-PLS, ANN, and MCR-ALS were developed and utilized for the quantitative assay of RNV and NMV in different matrices. Employing multiple, advanced multivariate models achieved accurate and efficient analysis of these drug combinations. A comprehensive comparison between the models was held where a reduction in the prediction error reflects the enhanced generalization of the ANN and MCR-ALS models, ensuring their better performance when analyzing future batches. The predictive power of the PLS model was further assessed using the GA tool for variable selection. GA successfully optimized the spectral dataset while minimizing redundancy. The evolutionary process demonstrated stable convergence, improved variable selection accuracy, and enhanced predictive performance. By integrating these selection strategies, GA effectively mitigated local optimization challenges, leading to more reliable spectral analysis. Despite the remaining consistency of the LVs as determined in the original PLS model, the resulting RMSEC values were lower than those of the PLS model indicating better model accuracy with reduced data dimensionality. The hybrid GA-ICOMP-PLS method was utilized to enhance the models’ predictive powers. Prediction error values of 0.15 and 0.14, and r values of 0.9997 and 0.9998 were obtained for RNV and NMV, respectively, indicating strong predictive power and generalization. The Savitzky-Golay filter, spectral derivatization, and SNV transformation were also employed to enhance the PLS spectral data signals. Low RMSEC values of 0.19 for both RNV and NMV were attained, indicating minimal scattered residuals and higher model performance. Additionally, the model’s predictive capabilities were enhanced using systematic LHS validation strategies. The latter provided a more robust and diverse dataset by capturing the underlying variabilities across space dimensions thus avoiding biased sampling. Integrating LHS strategies significantly enhanced the consistency and reliability of the developed chemometric models, enabling competent performance across diverse fields. Greenness, whiteness, and blueness studies were implemented using SPMS, AGREEprep, AGREE, RGB12, and BAGI metrics besides the ACS GCIPR and the SUSSOL solvent selection tools. These implemented metrics revealed the high sustainability, wide practicality, and eco-friendliness of the developed models. The key outcomes of the current study were effectively achieved where (i) the successful integration of LHS was highlighted, (ii) the development and comparison of models’ performance demonstrated their suitability for real-world applications, (iii) advanced signal preprocessing techniques were used to improve signal quality and model interpretability, (iv) evaluation of dataset homogeneity using PCA score plots confirmed that the LHS-generated datasets were well-distributed and suitable for multivariate modeling and (v) environmental and practical assessment of the models confirmed their environmentally sustainable, economically viable, and analytically powerful.

## Electronic supplementary material

Below is the link to the electronic supplementary material.

Supplementary Material 1

## Data Availability

The authors declare that the data supporting the findings of this study are available within the paper and its Supplementary Information files. Should any data files be needed in another format they are available from the corresponding author upon reasonable request.
