# Supplementary material for: Sustainable analysis of COVID-19 Co-packaged paxlovid: exploring advanced sampling techniques and multivariate processing tools
Source: BMC Chem. 2025 Jul 10;19(1):206. doi: 10.1186/s13065-025-01567-2 (PMC12243336; doi:10.1186/s13065-025-01567-2)
Supplement: Supplementary file 1 — Supplementary Material 1 [file 13065_2025_1567_MOESM1_ESM.docx]

**Supplementary information for**

**“Sustainable Analysis of COVID-19 Co-packaged Paxlovid: Exploring Advanced Sampling Techniques and Multivariate Processing Tools"**

Shymaa S. Soliman ^a,1*^, Nisreen F Abo Talib ^b,2^, Mohamed R. Elghobashy ^c,a,3^, Mona A. Abdel Rahman ^a,4^

^a^ Analytical Chemistry Department, Faculty of Pharmacy, October 6 University, October 6 City, Giza, 12858, Egypt.

^b^ Egyptian Drug Authority, Agouza, Giza, P.O. Box 35521, Egypt.

^c^ Analytical Chemistry Department, Faculty of Pharmacy, Cairo University, El-Kasr-El Aini Street, Cairo, 11562, Egypt.

* Corresponding author email: [shimaasayed@o6u.edu.eg](mailto:shimaasayed@o6u.edu.eg)

* Mailing address: October 6 University, Faculty of Pharmacy, October 6 City, Giza, 12858, Egypt.

* Tel: +20-1004581866

^1^ ORCID ID: 0000-0002-0554-0423

^2^ ORCID ID: 0000-0003-3869-8574

^3^ ORCID ID: 0000-0003-0383-2091

^4^ ORCID ID: 0009-0003-0978-469X

## Assessment of solvents' greenness

Appropriate selection of different solvents can significantly minimize the environmental hazards of different analytical processes while reducing the associated health risks, supporting compliance with sustainability goals and regulatory standards. Common solvents such as water, methanol, ethanol, and acetonitrile were evaluated to identify the most suitable solvent for this application. To ensure a sustainable choice, two evaluation tools were implemented such as the American Chemical Society Green Chemistry Institute Pharmaceutical Roundtable solvent selection tool (ACS GCIPR tool) and the Sustainable Solvents Selection tool (SUSSOL tool). These tools are specifically designed to facilitate decision-making by providing comprehensive data on solvent properties and detailed sustainability estimations. Besides ranking solvents based on greenness, these tools provide sufficient insights into their toxicity, biodegradability, energy efficiency, and ecological impact. Implementing these tools enhances the performance, practicality, and environmental responsibility of analytical processes.

### Solvent selection tool of the ACS GCIPR

The ACS GCIPR solvent selection tool encourages the use of greener solvents in pharmaceutical and chemical processes where the solvents are evaluated based on three criteria such as safety, health, and environmental profiles related to air, water, and waste (SHE), each scored on a scale of 1 to 10 [1]. The tool classifies solvents into three categories; Recommended, Usable, and Problematic according to their toxicity, flammability, and environmental persistence, aiding in identifying sustainable alternatives while reducing the use of hazardous substances. Additionally, the tool offers detailed information regarding solvent properties such as boiling point, polarity, biodegradability, and bioaccumulation possibilities thus supporting decision-making and sustainability optimization without affecting method performance.

### Sustainable solvent selection tool (SUSSOL)

The SUSSOL tool is a user-friendly software that was developed by Hannes Sels and his colleagues using Artificial Intelligence (AI) capabilities [2]. It functions by clustering solvents’ databases according to their physical properties where the solvents are processed using a neural network of a self-organized map (SOM) from Kohonen and visually represented in SUSSOL software. A multidimensional scaling mode (MDS) was then used to measure the similarities and dissimilarities between used solvents, providing deeper insights into their comparative characteristics. The results of the SUSSOL tool are visualized using a radar plot and bar chart. The radar plot compares the solvents’ sustainability parameters such as toxicity, biodegradability, volatility, and energy consumption. Each solvent’ performance is displayed as a line with filled areas that represent the value of each parameter, allowing for a quick visual comparison between solvents. Complementary, the bar chart compares the numerical scores of each solvent across these sustainability parameters, offering a detailed evaluation of each solvent's performance.

**Supplementary material (Table S1).** Concentrations of ritonavir and nirmatrelvir in the calibration and validation sets using different sampling techniques.

|  | **Calibration set** | | **Validation set** | | | |
| --- | --- | --- | --- | --- | --- | --- |
|  |  |  | **Latin Hypercube sampling** | | **Monte Carlo sampling** | |
| **Mixture No.** | **Concentrations (µg mL^-1^)** | | **Concentrations (µg mL^-1^)** | | **Concentrations (µg mL^-1^)** | |
|  | **RNV** | **NMV** | **RNV** | **NMV** | **RNV** | **NMV** |
| 1 | 15.00 | 15.00 | 17.50 | 13.00 | 21.29 | 14.71 |
| 2 | 15.00 | 5.00 | 17.10 | 19.60 | 23.12 | 21.01 |
| 3 | 5.00 | 5.00 | 6.30 | 5.70 | 7.54 | 7.84 |
| 4 | 5.00 | 25.00 | 19.50 | 7.20 | 23.27 | 13.44 |
| 5 | 25.00 | 10.00 | 12.70 | 17.50 | 17.65 | 23.31 |
| 6 | 10.00 | 25.00 | 7.10 | 13.90 | 6.95 | 20.84 |
| 7 | 25.00 | 15.00 | 12.60 | 24.40 | 10.57 | 24.19 |
| 8 | 15.00 | 10.00 | 12.00 | 10.40 | 15.94 | 18.11 |
| 9 | 10.00 | 10.00 | 23.80 | 12.40 | 24.15 | 5.71 |
| 10 | 10.00 | 20.00 | 24.50 | 22.90 | 24.30 | 21.98 |
| 11 | 20.00 | 25.00 | 6.70 | 23.20 | 8.15 | 23.68 |
| 12 | 25.00 | 20.00 | 22.00 | 18.50 | 24.41 | 18.57 |
| 13 | 20.00 | 15.00 | 22.90 | 6.50 | 24.14 | 20.15 |
| 14 | 15.00 | 25.00 |  | | | |
| 15 | 25.00 | 25.00 |  |  |  |  |
| 16 | 25.00 | 5.00 |  |  |  |  |
| 17 | 5.00 | 20.00 |  |  |  |  |
| 18 | 20.00 | 5.00 |  |  |  |  |
| 19 | 5.00 | 15.00 |  |  |  |  |
| 20 | 15.00 | 20.00 |  |  |  |  |
| 21 | 20.00 | 20.00 |  |  |  |  |
| 22 | 20.00 | 10.00 |  |  |  |  |
| 23 | 10.00 | 5.00 |  |  |  |  |
| 24 | 5.00 | 10.00 |  |  |  |  |
| 25 | 10.00 | 15.00 |  |  |  |  |

^*^ RNV: ritonavir, NMV: nirmatrelvir.

**Supplementary material (Table S2).** Configuration of the Genetic Algorithm parameters.

| **Parameter** | **Value** |
| --- | --- |
| Population size | 50 |
| Maximum generations | 100 |
| Mutation rate | 0.006 |
| The number of variables in a window (window width) | 4 |
| Percent of population (% of convergence) | 100 |
| % Wavelengths used at initiation | 40 |
| Crossover type | Single |
| Maximum number of latent variables | 5 |
| Cross-validation | Random |
| Number of subsets to divide data into for cross-validation | 5 |
| Number of iterations for cross-validation at each generation | 4 |

**Supplementary material (Table S3).** Validation set analysis using the established chemometric models.

| **Concentrations (μg mL^-1^)** | | **PLS** | | **GA-PLS** | | **ANN** | | **MCR-ALS** | |
| --- | --- | --- | --- | --- | --- | --- | --- | --- | --- |
|  |  | **Recovery %** | | **Recovery %** | | **Recovery %** | | **Recovery %** | |
| **RNV** | **NMV** | **RNV** | **NMV** | **RNV** | **NMV** | **RNV** | **NMV** | **RNV** | **NMV** |
| 17.50 | 13.00 | 98.83 | 99.49 | 99.93 | 99.10 | 100.69 | 98.50 | 99.985 | 100.32 |
| 17.10 | 19.60 | 98.81 | 99.71 | 99.02 | 97.78 | 100.19 | 97.91 | 100.53 | 100.56 |
| 6.30 | 5.70 | 101.72 | 97.19 | 97.23 | 99.44 | 99.32 | 100.32 | 99.524 | 99.86 |
| 19.50 | 7.20 | 98.47 | 98.49 | 97.68 | 97.12 | 99.47 | 99.42 | 98.96 | 100.99 |
| 12.70 | 17.50 | 97.82 | 101.23 | 100.31 | 101.18 | 100.90 | 99.36 | 99.12 | 99.37 |
| 7.10 | 13.90 | 101.44 | 101.50 | 98.53 | 101.60 | 101.51 | 101.02 | 101.02 | 100.65 |
| 12.60 | 24.40 | 98.32 | 100.62 | 100.79 | 99.97 | 96.70 | 98.86 | 99.81 | 99.35 |
| 12.00 | 10.40 | 98.08 | 101.42 | 101.09 | 99.37 | 98.24 | 97.95 | 99.30 | 100.43 |
| 23.80 | 12.40 | 97.32 | 101.92 | 101.34 | 99.29 | 97.86 | 101.00 | 99.04 | 98.37 |
| 24.50 | 22.90 | 97.66 | 98.40 | 100.21 | 100.84 | 97.94 | 100.91 | 100.29 | 99.08 |
| 6.70 | 23.20 | 101.97 | 100.88 | 96.99 | 99.51 | 99.61 | 100.58 | 100.77 | 100.18 |
| 22.00 | 18.50 | 97.23 | 101.88 | 101.09 | 98.64 | 98.51 | 100.32 | 101.28 | 98.49 |
| 22.90 | 6.50 | 101.25 | 101.09 | 99.80 | 99.27 | 97.74 | 99.33 | 98.96 | 100.39 |
| **Mean** | | 99.15 | 100.29 | 99.54 | 99.47 | 99.13 | 99.65 | 99.89 | 99.85 |
| **RSD%** | | 1.787 | 1.504 | 1.521 | 1.260 | 1.451 | 1.128 | 0.822 | 0.841 |
| **RMSEP ^a^** | | 0.35 | 0.20 | 0.19 | 0.18 | 0.29 | 0.18 | 0.16 | 0.13 |

^a^ Root mean square error of prediction.

^*^ RNV: ritonavir, NMV: nirmatrelvir.

**Supplementary material (Figure S1).** Predicted versus actual concentrations of ritonavir and nirmatrelvir using a PLS regression model based on Savitzky-Golay smoothing combined with standard normal variate (SNV) and derivative preprocessing. (a) original unprocessed data (b) First derivative combined with Savitzky-Golay and SNV preprocessing; (c) Second derivative combined with Savitzky-Golay and SNV preprocessing. The red dashed line represents the ideal regression line.

**
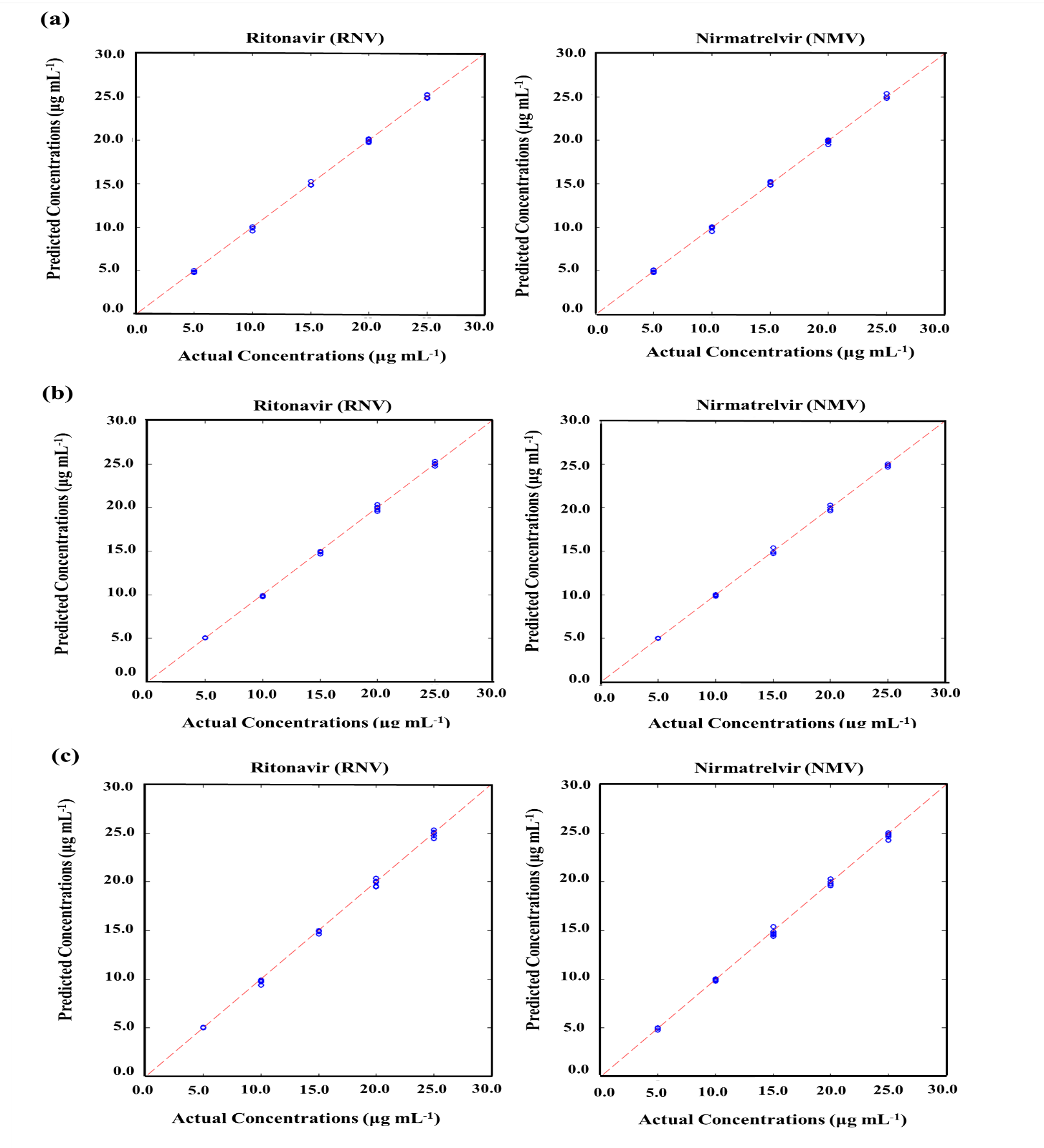
**

**Supplementary material (Figure S2).** Principal Component Analysis score plot of the first two principal components for the calibration (black-edged) and validation (orange-edged) sets of ritonavir and nirmatrelvir. The color gradient represents the total drug concentration. The plot illustrates homogeneity between sets and concentration-driven variance, confirming the dataset's suitability for multivariate calibration.

**
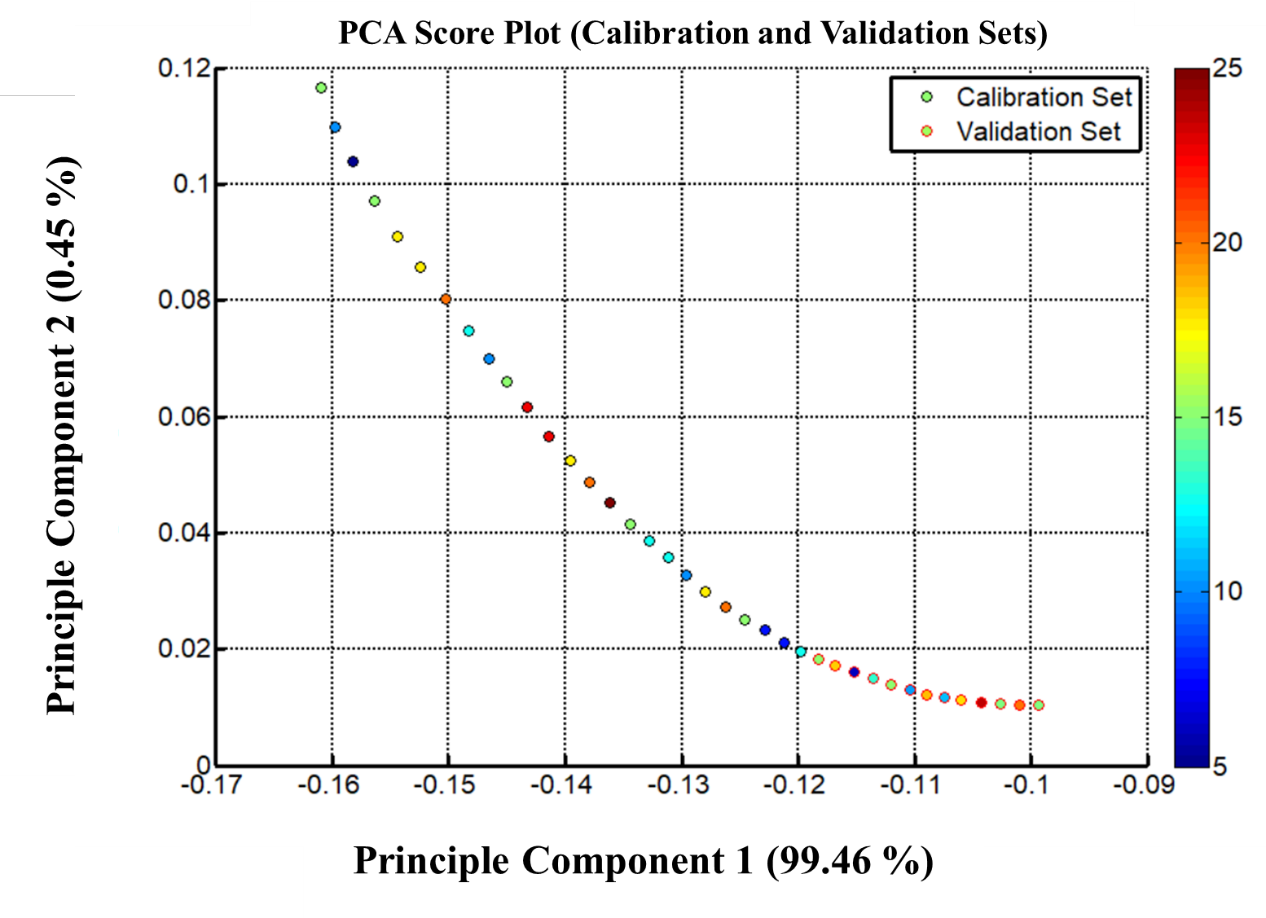
**

**Supplementary material (Figure S3).** Even distribution of calibration sample concentrations for both ritonavir and nirmatrelvir using a multilevel multifactorial design.

**
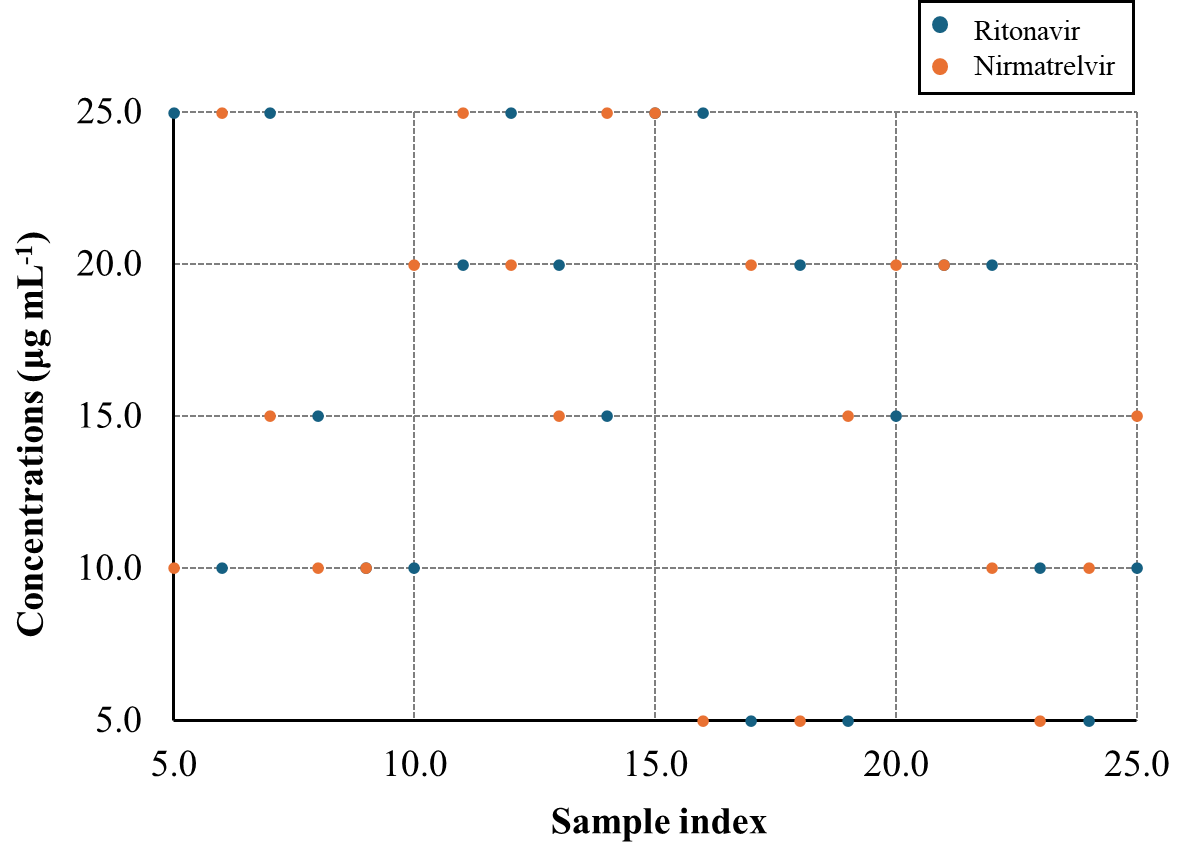
**

**Supplementary material (Figure S4).** RMSECV plot of the cross-validation results of the calibration set as a function of the optimum latent variables used to construct (a) the PLS model and (b) the GA-PLS model.


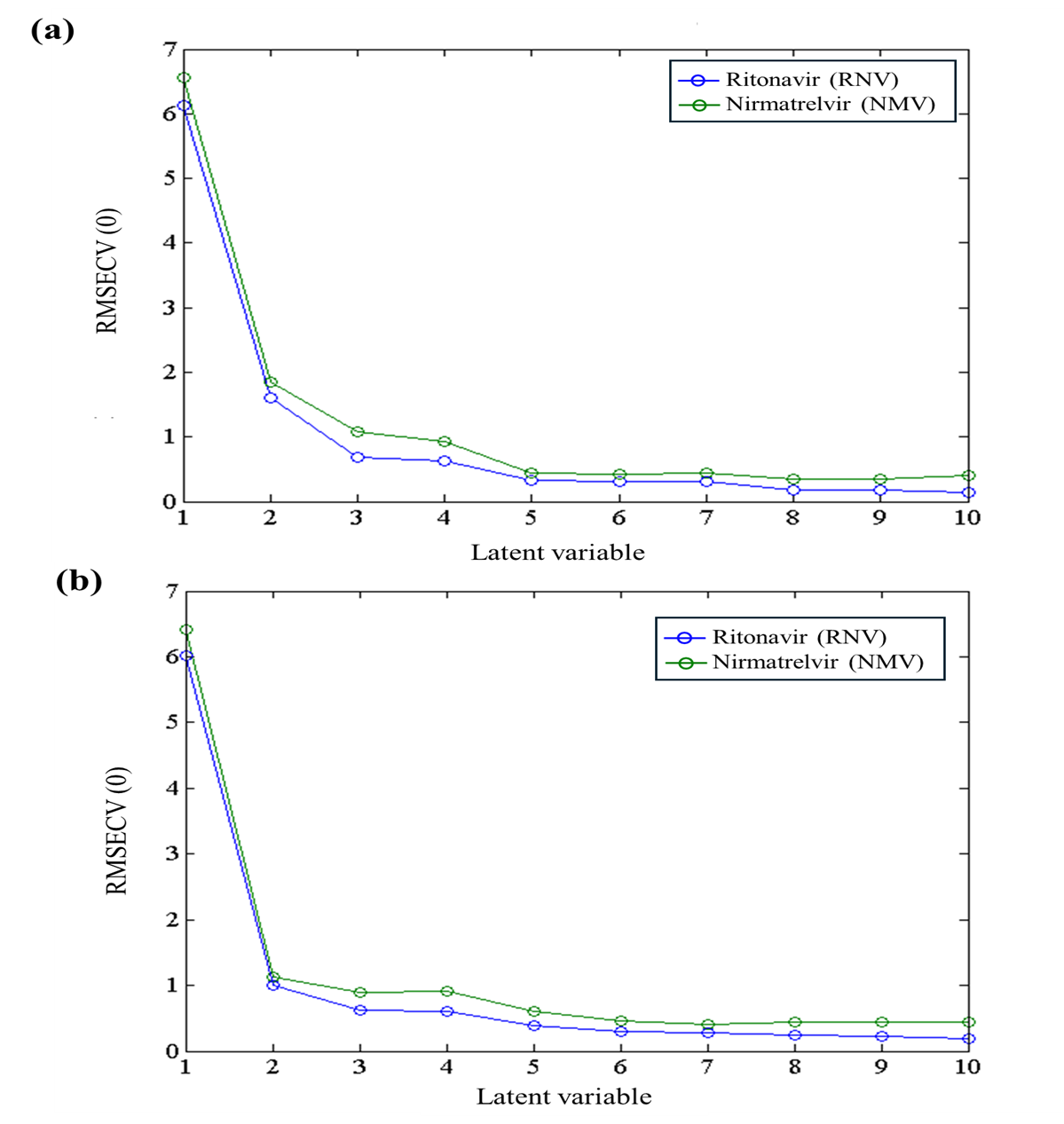


**Supplementary material (Figure S5).** Performance of the genetic algorithm parameters over 100 generations where (a) fitness versus number of windows at final generation, (b) evolution of average and best fitness, (c) average number of windows used, and (d) frequency of window inclusion.


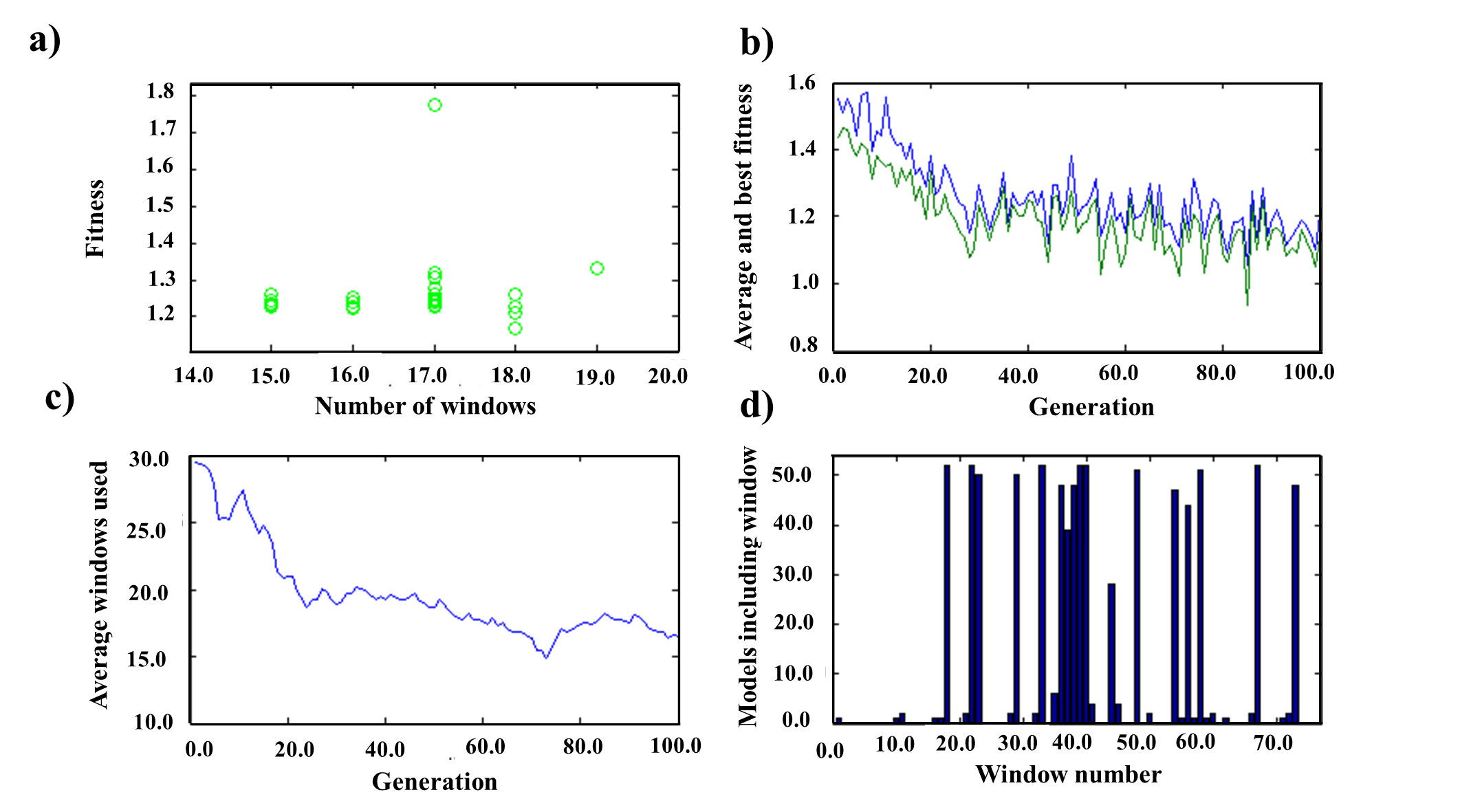


**Supplementary material (Figure S6).** (a) The RMSEP and (b) the relative standard deviation values calculated using the external validation data set for the two drugs using the developed models

**
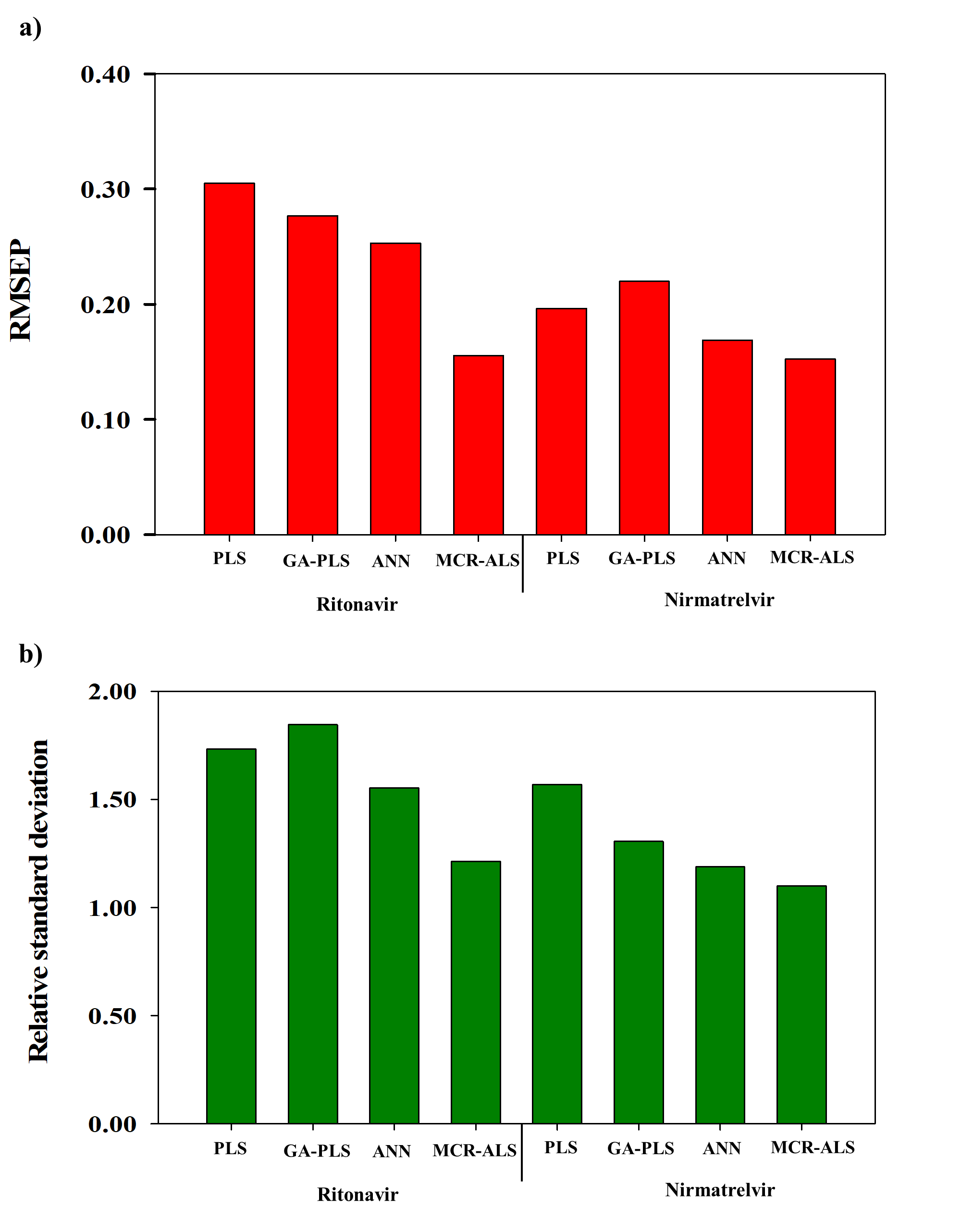
**

**Supplementary material (Figure S7).** Comparison of the sustainability profiles of the proposed and reported methods using the assessment tools.

| **Reference** | **Greenness evaluation** | **Reference** | **Greenness evaluation** |
| --- | --- | --- | --- |
| Proposed method | 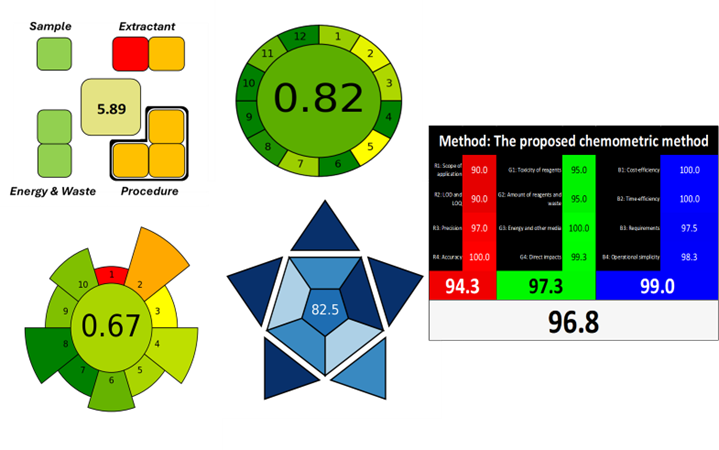 | | |
| [3] | 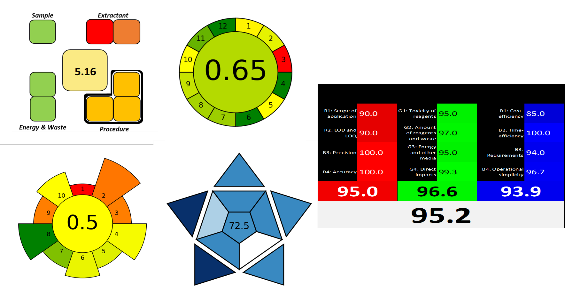 | [4] | 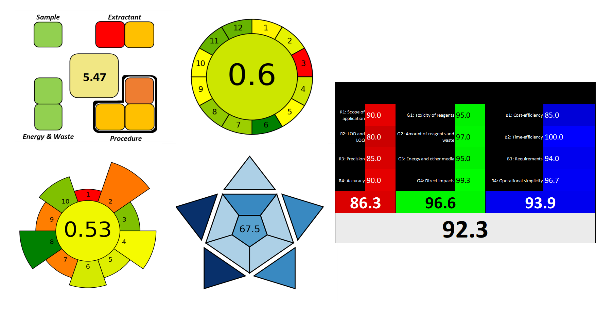 |
| [5] | 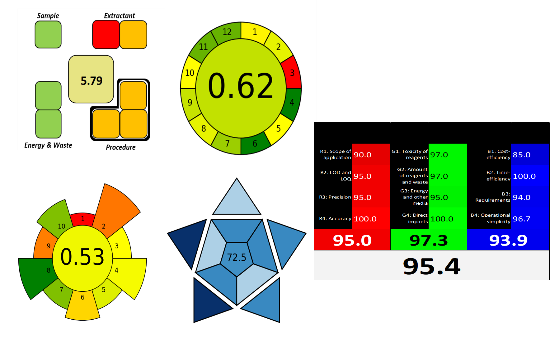 | [6] | 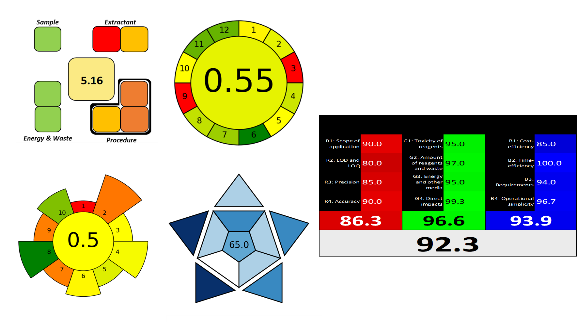 |
| [7] | 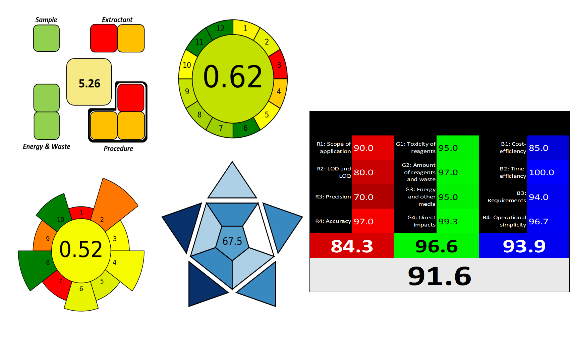 | [8] | 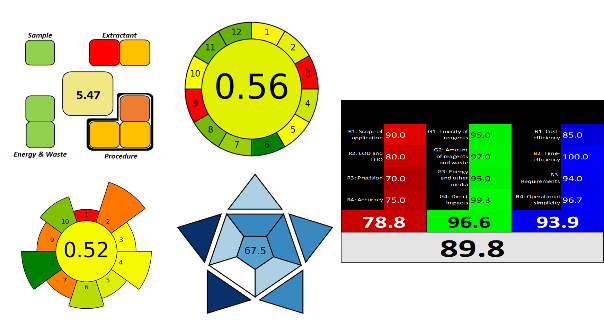 |
| [9] | 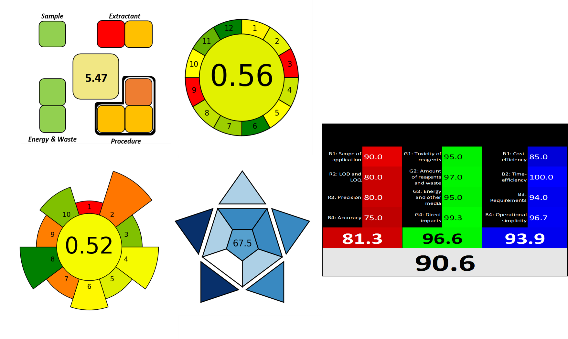 | [10] | 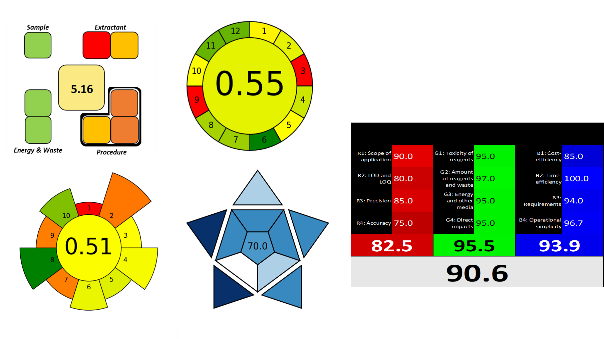 |

**Continue supplementary material (Figure S7).** Comparison of the sustainability profiles of the proposed and reported methods using the assessment tools.

| **Reference** | **Greenness evaluation** | **Reference** | **Greenness evaluation** |
| --- | --- | --- | --- |
| [11] | 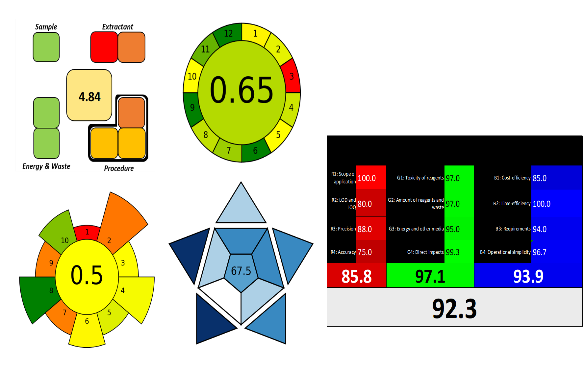 | [12] | 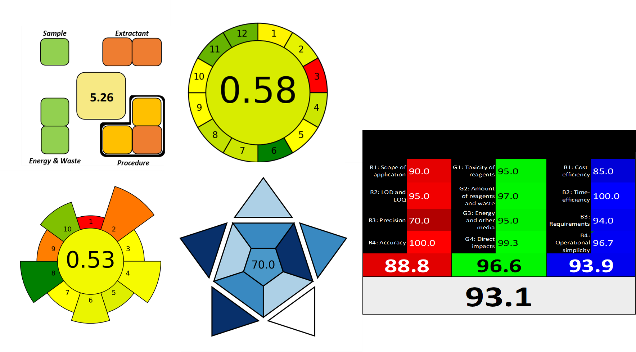 |
| [13] | 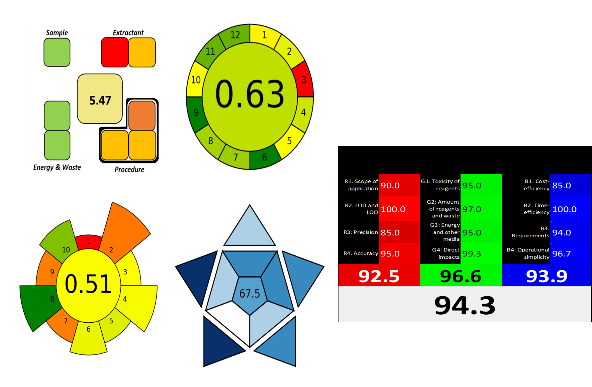 | [14] | 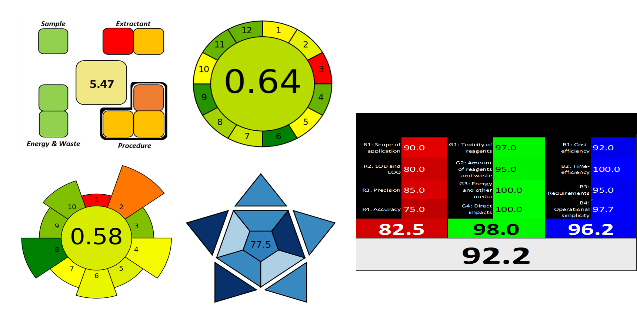 |
| [15] | 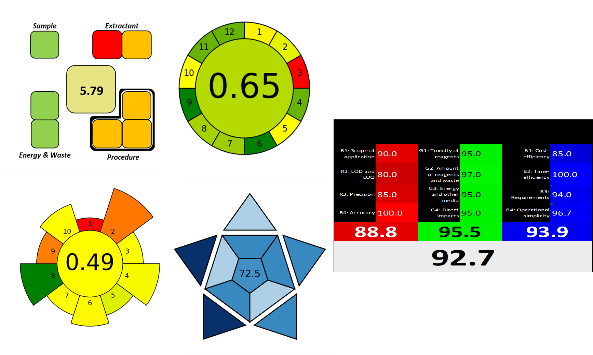 | [16] | 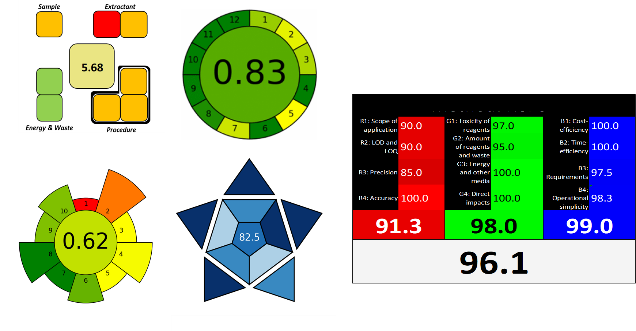 |
| [17] | 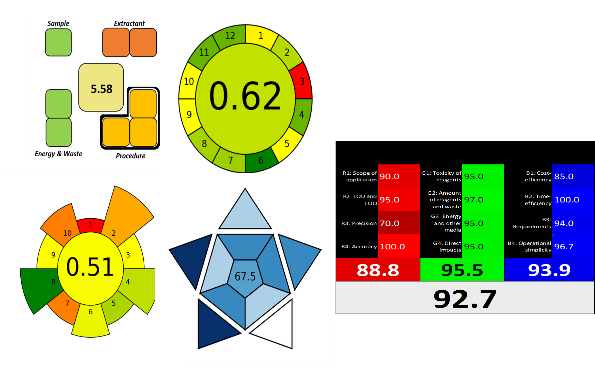 |  | |

**References:**

1. Diorazio LJ, Hose DRJ, Adlington NK. Toward a More Holistic Framework for Solvent Selection. Org Process Res Dev. 2016;20:760–73.

2. Sels H, De Smet H, Geuens J. SUSSOL-Using artificial intelligence for greener solvent selection and substitution. Molecules. 2020;25:3037.

3. Marella VL, Mandapaka V, Achanti S, Naseema M. A novel and robust analytical technique for determining covid-19 medications used in emergencies. NeuroQuantology. 2022;20:1717–25.

4. Zhao F, Xiang Z, Han J, Pan J, Qu Y, Fan K, et al. Simultaneous quantification of nirmatrelvir/ritonavir in human serum by LC–HRMS. J Pharm Biomed Anal. 2024;237:115796.

5. Imam MS, Batubara AS, Gamal M, Abdelazim AH, Almrasy AA, Ramzy S. Adjusted green HPLC determination of nirmatrelvir and ritonavir in the new FDA approved co-packaged pharmaceutical dosage using supported computational calculations. Sci Rep. 2023;13:137–46.

6. Martens-Lobenhoffer J, Böger CR, Kielstein J, Bode-Böger SM. Simultaneous quantification of nirmatrelvir and ritonavir by LC-MS/MS in patients treated for COVID-19. J Chromatogr B. 2022;1212:123510.

7. Abdallah IA, Hammad SF, Bedair A, Mansour FR. Homogeneous liquid–liquid microextraction coupled with HPLC/DAD for determination of nirmatrelvir and ritonavir as COVID-19 combination therapy in human plasma. BMC Chem. 2023;17:1–11.

8. Zhu X, Li L, Dai B, Liu Z, Wang Z, Cui L, et al. A simple and rapid LC-MS/MS method for the quantification of nirmatrelvir/ritonavir in plasma of patients with COVID-19. Int J Anal Chem. 2024;2024:1–9.

9. Liu C, Zhu M, Cao L, Boucetta H, Song M, Hang T, et al. Simultaneous determination of nirmatrelvir and ritonavir in human plasma using LC–MS/MS and its pharmacokinetic application in healthy Chinese volunteers. Biomed Chromatogr. 2022;36:1–9.

10. Xu Z, Li C, Qian X, Duan H, Zhou J, Zhang Q, et al. A validated LC-MS/MS method for determination of six Anti-SARS-CoV-2 drugs in plasma and its application for a pharmacokinetic study in rats. J Chromatogr B. 2024;1235:124038.

11. Guyon J, Novion M, Fulda V, Ducint D, Molimard M, Couzi L, et al. A UPLC-MS/MS method for plasma biological monitoring of nirmatrelvir and ritonavir in the context of SARS-CoV-2 infection and application to a case. J Am Soc Mass Spectrom. 2022;33:1975–81.

12. Elbordiny HS, Alzoman NZ, Maher HM, Aboras SI. Tailoring two white chromatographic platforms for simultaneous estimation of ritonavir-boosted nirmatrelvir in their novel pills: degradation, validation, and environmental impact studies. RSC Adv. 2023;13:26719–31.

13. Zhou C-J, Liu Y, Wang A, Wu H, Xu R, Zhang Q. Simultaneous measurement of COVID-19 treatment drugs (nirmatrelvir and ritonavir) in rat plasma by UPLC-MS/MS and its application to a pharmacokinetic study. Heliyon. 2024;10:1–9.

14. Imam MS, Abdelazim AH, Ramzy S, Almrasy AA, Gamal M, Batubara AS. Higher sensitive selective spectrofluorometric determination of ritonavir in the presence of nirmatrelvir: application to new FDA approved co-packaged COVID-19 pharmaceutical dosage and spiked human plasma. BMC Chem. 2023;17:120–8.

15. Pallavi S, Sowjanya G. Development and validation of a new RP-UPLC method for the simultaneous estimation of nirmatrelvir and ritonavir in bulk and copacked tablet dosage forms. Res J Pharm Technol. 2023;16:4370–6.

16. Aboras SI, Maher HM, Alzoman NZ, Elbordiny HS. Sustainable and technically smart spectrophotometric determination of PAXLOVID: a comprehensive ecological and analytical performance rating. BMC Chem. 2024;18:184–200.

17. Imam MS, Abdelazim AH, Batubara AS, Gamal M, Almrasy AA, Ramzy S, et al. Simultaneous green TLC determination of nirmatrelvir and ritonavir in the pharmaceutical dosage form and spiked human plasma. Sci Rep. 2023;13:1–10.
